# scMAE: a masked autoencoder for single-cell RNA-seq clustering

**DOI:** 10.1093/bioinformatics/btae020

**Published:** 2024-01-16

**Authors:** Zhaoyu Fang, Ruiqing Zheng, Min Li

**Affiliations:** School of Computer Science and Engineering, Central South University, 932 South Lushan Road, Yuelu District, Changsha 410083, China; School of Computer Science and Engineering, Central South University, 932 South Lushan Road, Yuelu District, Changsha 410083, China; School of Computer Science and Engineering, Central South University, 932 South Lushan Road, Yuelu District, Changsha 410083, China

## Abstract

**Motivation:**

Single-cell RNA sequencing has emerged as a powerful technology for studying gene expression at the individual cell level. Clustering individual cells into distinct subpopulations is fundamental in scRNA-seq data analysis, facilitating the identification of cell types and exploration of cellular heterogeneity. Despite the recent development of many deep learning-based single-cell clustering methods, few have effectively exploited the correlations among genes, resulting in suboptimal clustering outcomes.

**Results:**

Here, we propose a novel masked autoencoder-based method, scMAE, for cell clustering. scMAE perturbs gene expression and employs a masked autoencoder to reconstruct the original data, learning robust and informative cell representations. The masked autoencoder introduces a masking predictor, which captures relationships among genes by predicting whether gene expression values are masked. By integrating this masking mechanism, scMAE effectively captures latent structures and dependencies in the data, enhancing clustering performance. We conducted extensive comparative experiments using various clustering evaluation metrics on 15 scRNA-seq datasets from different sequencing platforms. Experimental results indicate that scMAE outperforms other state-of-the-art methods on these datasets. In addition, scMAE accurately identifies rare cell types, which are challenging to detect due to their low abundance. Furthermore, biological analyses confirm the biological significance of the identified cell subpopulations.

**Availability and implementation:**

The source code of scMAE is available at: https://zenodo.org/records/10465991.

## 1 Introduction

Single-cell RNA sequencing (scRNA-seq) has revolutionized our understanding of cellular biology by enabling gene expression analysis at the individual cell level. This breakthrough technology has provided unprecedented insights into cellular heterogeneity, revealing previously unknown functional roles and driving advancements in studying the tumor microenvironment and advancing targeted therapies and personalized medicine (Buettner *et al.* 2015, Papalexi and Satija 2018). As a result, accurate identification of cell types has become a critical step in scRNA-seq analysis. Unsupervised clustering has been proven to be the most effective method for cell type identification, as it can automatically discover similarities and differences between cells, unravel cell heterogeneity, and explore the intrinsic structure and patterns of the data without relying on prior knowledge (Kiselev *et al.* 2019, Zhao *et al.* 2024). By grouping cells based on gene expression patterns, clustering facilitates the exploration of distinct cell populations, enabling further analysis and enhancing our understanding of cellular biology and disease mechanisms.

Currently, there are several clustering methods for single-cell analysis (Qi *et al.* 2020, Zhao *et al.* 2023). The first category involves dimensionality reduction of the gene expression matrix, followed by the use of traditional clustering methods such as k-means or hierarchical clustering for clustering. For example, pcaReduce (Žurauskienė and Yau 2016) combines principal component analysis (PCA) with k-means clustering, associating each cluster branch with a principal component variable to obtain hierarchical clustering results. CIDR (Lin *et al.* 2017) performs data imputation on gene expression data and then uses the first few principal coordinates of the imputed data for hierarchical clustering. The second category of methods is based on graphs. Seurat (Satija *et al.* 2015) utilizes a shared nearest neighbor (SNN) graph to describe the similarity between cells and performs clustering using the Louvain (Blondel *et al.* 2008) algorithm. The construction of the SNN graph is also based on the principal components of the gene expression matrix. However, PCA is a linear dimensionality reduction method that may not capture the complex nonlinear relationships between genes. The third category comprises ensemble methods. SC3 (Kiselev *et al.* 2017) integrates results from multiple clustering algorithms to achieve more stable and consistent clustering results. SIMLR (Wang *et al.* 2017) combines multiple kernel functions with a multi-kernel Bayesian learning algorithm during the dimensionality reduction process to capture different features and relationships in the data. RCSL (Mei *et al.* 2021) constructs a similarity matrix by measuring both global and local relationships between cells and then derives a block diagonal matrix from it to obtain the final clustering results. SMSC (Qi *et al.* 2021) adopts a multiple kernel combination approach, enabling direct learning of similarity metrics from single-cell RNA sequencing data while simultaneously considering the constraints of clustering structure, thereby discovering effective cell clusters.

In recent years, deep embedding clustering methods have been successfully applied to high-dimensional and sparse single-cell RNA sequencing data (Wang *et al.* 2022). Among these methods, autoencoders, a popular type of self-supervised deep neural networks, stand out for their proficiency in learning compact representations of high-dimensional data (Tschannen *et al.* 2018). Eraslan *et al.* (2019) proposed the deep count autoencoder (DCA) model based on the zero-inflated negative binomial (ZINB) distribution for denoising and low-dimensional representation learning. The DCA model takes the raw count expression matrix as input, reduces noise using an encoder, maps the input data to a low-dimensional space, and reconstructs the original count data distribution using a decoder. The model uses the negative log-likelihood as the loss function and outputs parameters related to the negative binomial distribution, such as mean, dispersion, and dropout probability. After obtaining the low-dimensional representations, traditional clustering methods can be applied. scDeepCluster (Tian *et al.* 2019) integrates the DCA model with the deep embedding clustering (DEC) algorithm using the Kullback–Leibler divergence, achieving coordinated optimization of clustering and dimensionality reduction. The scziDesk (Chen *et al.* 2020a) model further emphasizes the concentration of similar cell types and adopts a weighted soft K-means clustering algorithm in the latent space. scVI (Lopez *et al.* 2018) uses variational inference to approximate the zero-inflated negative binomial distribution, effectively managing gene expression values. Svensson *et al.* (2020) improved scVI, enhancing model interpretability without a significant loss in accuracy. However, these methods do not effectively capture the correlations between genes as they simply add simple Gaussian noise or no noise to the inputs.

The second category of methods is based on graph neural networks. scGNN (Wang *et al.* 2021) constructs a cell–cell graph using the gene expression matrix and iteratively builds connections between cells using three multimodal autoencoders. On the other hand, graph-sc (Ciortan and Defrance 2022) utilizes a gene–cell graph, treating both cells and genes as graph nodes. Unlike the cell–cell graph, there are no direct connections between cells in graph-sc, only connections between genes and cells. Cell embeddings for clustering are obtained using graph autoencoders. However, since scRNA-seq data only consists of gene expression data, both the cell–cell graph and the gene–cell graph are constructed based on the gene expression matrix. As a result, the graph structure information and the node feature information essentially represent the same information, limiting the full potential of graph neural networks.

The third category is based on contrastive learning. However, applying existing methods originally designed for image and language data to single-cell data is challenging because these methods heavily rely on spatial or semantic features. Both contrastive-sc (Ciortan and Defrance 2021), CLEAR (Han *et al.* 2022), and scNAME (Wan *et al.* 2022) are all methods based on contrastive learning methods. contrastive-sc creates augmented samples by masking some genes of cells. However, simply randomly zeroing out or adding Gaussian noise to some features may not effectively capture important patterns between features. CLEAR, on the other hand, performs more complex operations such as swapping gene values between two cells to create augmented samples. scNAME constructs positive sample pairs based on the nearest neighbors of gene expression. The contrastive loss used in contrastive learning strengthens the similarity between positive sample pairs (i.e. a sample and its augmented sample) while increasing the distance between negative sample pairs (i.e. a sample and other samples) (Chen *et al.* 2020b). However, these methods may mistakenly treat cells belonging to the same cluster as negative sample pairs, resulting in false clustering results.

Significant advancements have been made in the field of natural language processing through the use of self-supervised learning techniques, including autoregressive language models like GPT (Radford *et al.* 2018) and masked autoencoder models like BERT (Devlin *et al.* 2018). These approaches involve masking a portion of the data and training the model to predict the masked content. By doing so, the resulting low-dimensional embeddings capture semantic information, contextual relationships, and syntactic structures within the text, effectively capturing the relationships between words and sentences. Similar strategies have also been applied in computer vision, such as masked autoencoders (MAE) (He *et al.* 2022), which mask random regions of input images and reconstruct the missing pixels.

Inspired by these methods, our study proposes scMAE, a masked autoencoder specifically designed for scRNA-seq data analysis. In scMAE, we randomly shuffle each gene with a certain probability and input them into the encoder to obtain low-dimensional representations. This shuffling of the input data serves the purpose of denoising and enables the model to learn the correlations between genes, resulting in more meaningful low-dimensional representations. A masking predictor is then employed to predict whether the expression values have been shuffled. Subsequently, the concatenated low-dimensional representations and masking prediction results are passed through the decoder to reconstruct the original gene expression matrix. The masking prediction results guide the decoder in identifying which gene values have been disrupted, facilitating a more accurate reconstruction of the original gene expression matrix. Experimental results on 15 real scRNA-seq datasets demonstrate the superior performance of scMAE in terms of clustering compared to state-of-the-art single-cell clustering methods. In addition, scMAE exhibits the ability to identify rare cell types, further highlighting its effectiveness.

## 2 Materials and methods

### 2.1 Method

The input is a processed gene expression matrix $X\in R^{C\times G}$ (see Supplementary Note S1 for details), where $X_{ij}$ represents the expression of the *j*th gene in the *i*th cell, *C* is the number of cells, *G* is the number of genes. The objective of scMAE is to train a model that can estimate the mask vector applied to each cell and subsequently reconstruct the original gene expression from its corrupted version.

#### 2.1.1 Generation of masked gene expression matrix

To introduce variability and perturbation into the gene expression matrix, three steps are taken. Firstly, the gene expression values within each gene in the matrix *X* are randomly shuffled. This shuffling process involves permuting the order of expression values while maintaining the associations within each gene. The resulting shuffled gene expression matrix is denoted as $X^{\prime }$. Next, we generate a mask matrix *M* using the Bernoulli distribution. This mask matrix determines which elements of the gene expression matrix will be modified. It is generated based on a list of probabilities $\left(p_{1},p_{2},\ldots ,p_{G}\right)$, in which each probability corresponds to the likelihood of modifying the expression values of the respective gene. The mask matrix *M* is then generated by applying the Bernoulli distribution as follows:

(1) $$M_{ij}∼\mathrm{Bernoulli}(p_{j}),$$

where $M_{ij}$ denotes the element at the *i*th row and *j*th column of the mask matrix *M*, and $p_{j}$ represents the probability associated with the *j*th gene in the list of probabilities, controlling the proportion of the feature that will be masked and corrupted.

Finally, the masked gene expression $X_{M}$ is obtained by applying element-wise operations (Wan *et al.* 2022). The calculation can be expressed as:

(2) $$X_{M_{ij}}=X_{ij}\cdot (1-M_{ij})+X_{ij}^{\prime }\cdot M_{ij},$$

where $X_{ij}$ denotes the element at the *i*th row and *j*th column of the original gene expression matrix *X*, $X_{ij}^{\prime }$ represents the element at the *i*th row and *j*th column of the shuffled gene expression matrix $X^{\prime }$, and $X_{M_{ij}}$ represents the element at the *i*th row and *j*th column of the masked gene expression matrix $X_{M}$. By following these steps, the gene expression matrix *X* undergoes random shuffling of gene expression values within each gene and the application of a mask matrix, resulting in the modified gene expression matrix $X_{M}$.

#### 2.1.2 Masked autoencoder

**Figure 1.**
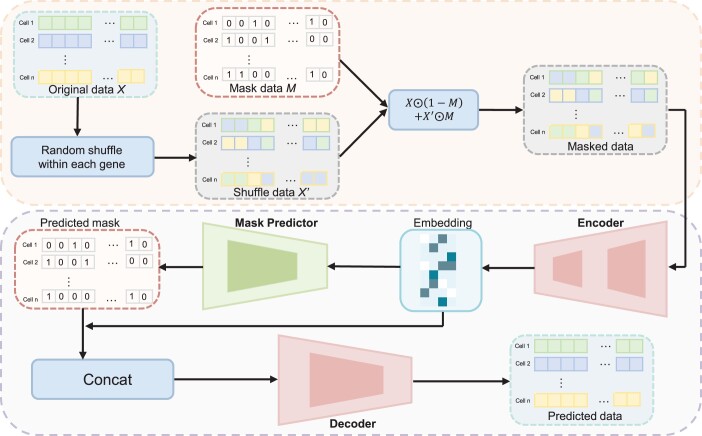
Workflow of scMAE. Initially, the expression matrix *X* undergoes a certain degree of shuffling to create a masked matrix, $X_{M}$. Next, $X_{M}$ is fed into the encoder, which captures the correlations among genes to generate low-dimensional cell embeddings. These embeddings are then inputted into a mask predictor to determine if masking was applied to the gene expression matrix during the first step. In the fourth step, the low-dimensional embeddings and vectors indicating masking status are concatenated and supplied to the decoder. Finally, these trained embeddings are employed for downstream cell clustering.

As illustrated in Fig. 1, the masked autoencoder consists of three components: an encoder, a mask predictor, and a decoder. The encoder maps the gene expression $X_{M}$ into a low-dimensional embedding *E*. Assuming the encoder has *L* layers, we denote the learned data representation from the *l*th layer as $E_{l}$, the weight matrix as $W_{l}$, and the bias vector as $b_{l}$. The input of the first layer is $E_{0}=X_{M}$. The learning process of the *l*th layer in the encoder is defined as follows:

(3) $$E_{l}=\sigma (W_{l}E_{l-1}+b_{l}),$$

where σ represents an activation function. The last layer of the encoder performs a linear transformation, meaning that σ is the identity function. Consequently, the output of the last layer $E_{l}$ corresponds to the embedding *E*.

For a modified gene expression $X_{M_{i.}}$ of a specific cell, it is possible that certain genes contain erroneous expression information, which poses challenges in directly reconstructing the original gene expression. Therefore, we divide the process into two steps. First, we utilize a mask predictor based on the latent embedding to identify which gene expression values are corrupted. Subsequently, the predicted results are fed into the decoder, which leverages the information about the corrupted genes and the latent embedding to reconstruct the gene expression. Having prior knowledge of the corrupted features greatly improves the efficiency of the reconstruction process. Specifically, the mask predictor utilizes the embedding *E* to predict whether the input $X_{M}$ is masked, and the resulting prediction is denoted as $\tilde{M}$. The mask predictor is implemented as a linear layer and is trained using the cross-entropy loss:

(4) $$L_{m}=-\sum_{ij}M_{ij}\;\text{log}({\tilde{M}}_{ij}),$$

where $M_{ij}$ represents the element at the *i*th row and *j*th column of the input mask *M*, while ${\tilde{M}}_{ij}$ denotes the element at the corresponding position in the predicted mask $\tilde{M}$. The loss function quantifies the discrepancy between the predicted mask and the true mask, with a higher penalty for incorrect predictions.

The decoder takes the concatenation of the embedding *E* and the predicted mask $\tilde{M}$ as input and maps it to the raw gene expression. Providing information about the corrupted inputs enables the decoder to reconstruct the raw gene expression more effectively. In scMAE, the decoder is implemented as a linear layer, allowing it to transform the concatenated input into the raw gene expression. In the reconstruction loss, we assign different weights to the corrupted and non-corrupted genes. We use a weighted mean squared error (MSE) loss function as follows:

(5) $$L_{r}=\frac{1}{N}\sum_{ij}\Omega_{ij}\cdot |X_{ij}-{\tilde{X}}_{ij}{|}^{2},$$

where ${\tilde{X}}_{ij}$ represents the element at the *i*th row and *j*th column of the decoder output $\tilde{X}$. The weight $\Omega_{ij}$ is calculated based on the binary mask $M_{ij}$ and a hyper-parameter λ, given by:

(6) $$\Omega_{ij}=M_{ij}\cdot \lambda +(1-M_{ij})\cdot (1-\lambda ).$$

The parameter λ determines the emphasis placed on the corrupted entries during the reconstruction process. By using the weighted MSE loss function, we aim to achieve a more accurate and comprehensive reconstruction of the original gene expression, giving higher importance to correctly reconstructing the genes affected by corruption.

In summary, the total loss function is calculated as:

(7) $$L=(1-\gamma )L_{r}+\gamma L_{m},$$

where $L_{r}$ is the mask weighted reconstruction loss, $L_{m}$ is the mask estimation loss and γ is a hyper-parameter that balances two losses. These two loss functions share the encoder. In the downstream clustering, we only rely on the cell embeddings generated by the encoder.

Based on our intuitive observations, we have concluded that the encoder’s ability to capture the correlations among the features of *X* and produce embeddings *E* can effectively reconstruct *X*. In this regard, the mask predictor identifies the masked features by detecting inconsistencies in the gene values. In addition, the decoder possesses prior knowledge about which features have been damaged. As a result, the decoder can focus on learning from the correlated non-masked features to fill in the missing information. The decoder’s awareness of the damaged features in advance is critical for achieving successful reconstruction. The learned cell representations, which capture the gene’s correlation, serve as informative embeddings for clustering.

#### 2.1.3 Clustering phase

After generating cell embeddings, we apply a clustering algorithm to assign cells to clusters. In this paper, we use two general clustering methods: K-means (Hartigan and Wong 1979) and Leiden (Traag *et al.* 2019) clustering. The choice between K-means and Leiden is based on the number of cells in the dataset. K-means is suitable for smaller datasets, while Leiden clustering is more effective for larger datasets. Specifically, we use the K-means method when the number of cells is <10000. For datasets with larger cell populations, we employ the Leiden method.

### 2.2 Datasets

The scMAE method is evaluated on a total of 16 real scRNA-seq datasets, each of which contains cells with known labels or validated in previous studies (Pollen *et al.* 2014, Macosko *et al.* 2015, Baron *et al.* 2016, Shekhar *et al.* 2016, Tirosh *et al.* 2016, Bach *et al.* 2017, Cao *et al.* 2017, Guo *et al.* 2018, Hrvatin *et al.* 2018, Tabula Muris Consortium *et al.* 2018, Tosches *et al.* 2018, Wang *et al.* 2018, Young *et al.* 2018, Tran *et al.* 2020). These datasets have been widely used for evaluating other clustering methods as well (Ciortan and Defrance 2021, Ciortan and Defrance 2022, Han *et al.* 2022, Wan *et al.* 2022, Yan *et al.* 2022). The characteristics of these datasets, including the biological tissues and organisms they represent, are summarized in Supplementary Table S1. The datasets cover a diverse range of tissues such as the cerebral cortex, mouse lung, mouse limb muscle, human kidneys, human testis, human pancreas, mouse spleen, and human fetal kidney, among others. The number of cells in these datasets varies from hundreds to tens of thousands, and the number of genes also varies accordingly. Among them, six datasets have cell numbers >18 000. In addition, the Spleen, Tosches, Guo, Baron, Shekhar, and Macosko datasets contain rare cell types that account for <0.5% of the total cells. These datasets were generated using different sequencing platforms such as Smart-seq2, 10x, sci-RNA-seq, inDrop, and Drop-seq, providing a comprehensive representation of scRNA-seq data generated by different technologies. To assess the impact of batch effects on these clustering methods, we also used a dataset MouseRetina, which contains batch effects.

### 2.3 Comparison methods

Seven state-of-the-art single-cell deep learning clustering methods, namely scNAME (Wan *et al.* 2022), scGNN (Wang *et al.* 2021), graph-sc (Ciortan and Defrance 2022), contrastive-sc (Ciortan and Defrance 2021), scVI (Lopez *et al.* 2018), scVI-LD (Svensson *et al.* 2020), and CLEAR (Han *et al.* 2022), were evaluated and compared. To ensure a fair comparison, each method underwent the pre-processing steps specified in its respective methodology. For CLEAR, the pre-processing involved normalization, log transformation, high variable gene selection, and scaling. These steps were performed following the online tutorial provided by the method’s authors (https://github.com/ml4bio/CLEAR). For contrastive-sc, graph-sc, and scNAME, the original data were used for subsequent pre-processing and clustering. The authors of these methods provided the necessary instructions for pre-processing the data. For both scVI and scVI-LD, their input consists of the original count matrix, with the selection focused on highly variable genes.

As for scGNN, data preprocessing and left truncated mixed Gaussian (LTMG) model processing are performed. These steps were carried out as specified in the method’s guidelines. In the evaluation, the number of clusters was provided for CLEAR, contrastive-sc, graph-sc, scVI, scVI-LD, and scNAME. However, scGNN does not require specifying the number of clusters; the method automatically identifies the number of clusters. Parameters for all methods were either set as default or adjusted following the guidelines provided by their respective authors.

### 2.4 Evaluation metrics

To conduct an effective evaluation of the performance of various clustering algorithms, we employ three widely utilized metrics: the Adjusted Rand Index (ARI) (Hubert and Arabie 1985), the Normalized Mutual Information (NMI) (Davies and Bouldin 1979), and the average silhouette width (ASW) (Rousseeuw 1987). ARI and NMI offer quantitative measures that evaluate the alignment between the derived clustering labels and the actual cell labels.

ARI quantifies the similarity between the predicted and true labels by considering all pairs of cells, thereby measuring the agreement beyond random chance. The ARI values span from −1 to 1, with 1 signifying a flawless clustering agreement, 0 indicating a random clustering result, and negative values suggesting a disagreement between the predicted and the true labels.

NMI, on the other hand, measures the mutual information between the clustering and the true labels while considering the distribution of labels and cluster assignments. Similar to ARI, the NMI values also range from 0 to 1, where 1 indicates perfect agreement in clustering.

The silhouette simultaneously considers the cohesion within clusters and the separation between clusters. Generally, an ASW score of 1 implies well-separated clusters, a score of 0 implies overlapping clusters and a score of -1 implies strong misclassification. Following Lotfollahi (Lotfollahi *et al.* 2022), we scale the ASW scores to a range between 0 and 1 using the formula:

(8) $$\text{cell-type}\mathrm{ASW}=\frac{\mathrm{ASW}+1}{2}.$$

Larger values correspond to denser clusters. We also calculate an ASW score on batches to assess the extent of batch effect removal. In this context, we scale and invert the ASW score for consistent metric comparison:

(9) BatchASW=1−abs(ASW)

A higher final score indicates better mixing, reflecting improved batch removal effects. By evaluating the clustering algorithms using these metrics, we can assess their effectiveness in accurately assigning cells to clusters and capturing the underlying structure of the scRNA-seq datasets.

## 3 Results

### 3.1 scMAE achieves excellent clustering performance and outperforms existing methods

To evaluate the effectiveness of scMAE, we compared it with seven deep learning methods, namely scNAME (Wan *et al.* 2022), scGNN (Wang *et al.* 2021), graph-sc (Ciortan and Defrance 2022), contrastive-sc (Ciortan and Defrance 2021), scVI (Lopez *et al.* 2018), scVI-LD (Svensson *et al.* 2020), and CLEAR (Han *et al.* 2022), on various datasets. Each method was run 10 times with different random seeds, and we presented the results using median values. The evaluation metrics employed include ARI, NMI, and cell-type ASW. ARI and NMI measure the similarity between the predicted clustering labels and the true labels. Meanwhile, cell-type ASW assesses the average silhouette width between different cell types.

**Figure 2.**
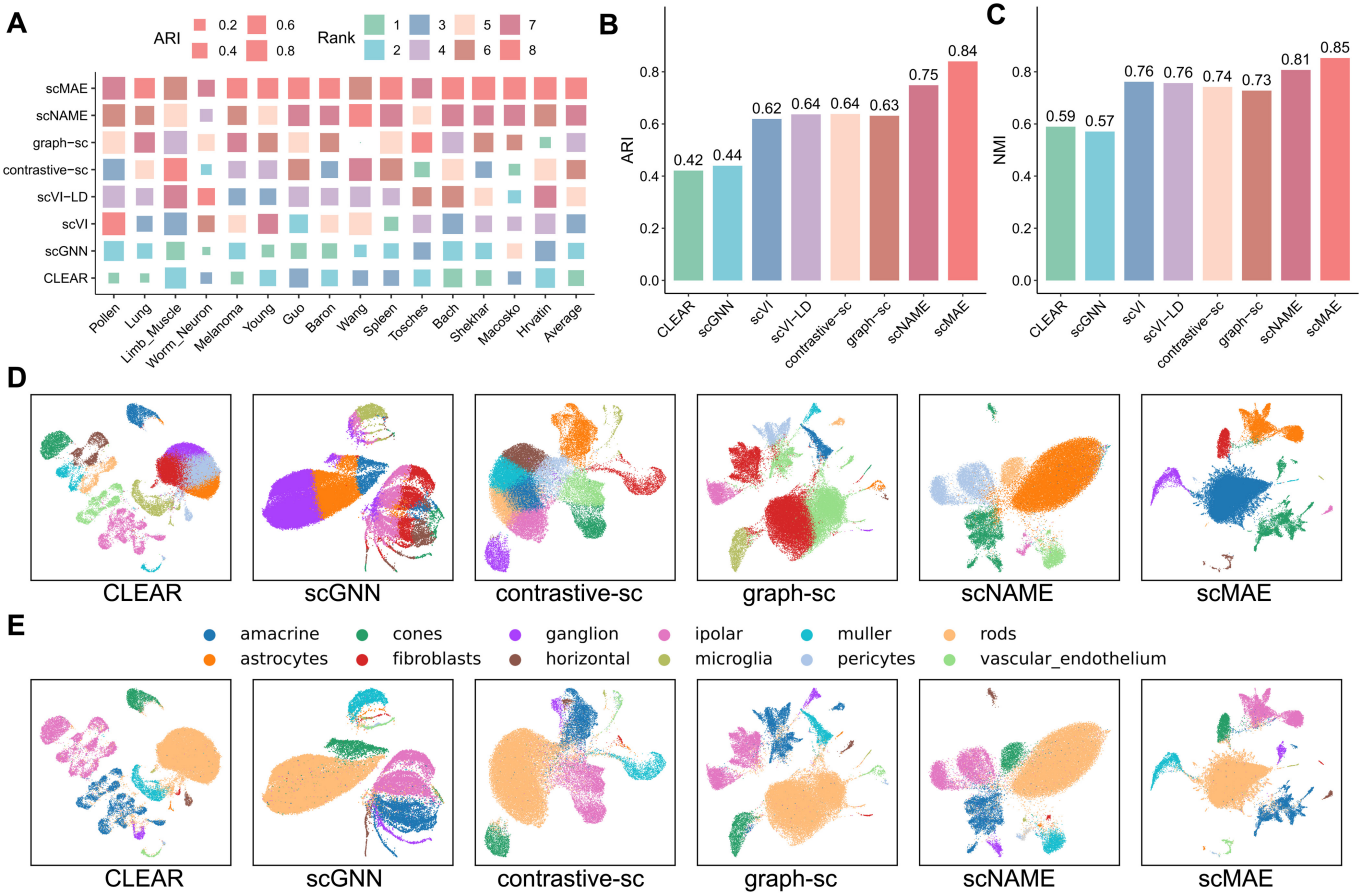
Real scRNA-seq data analysis results. (A) ARI scores of scMAE and seven comparative methods on 15 real scRNA-seq datasets. Each block represents the performance of a method on a dataset, where the size indicates the ARI score and the color represents the rank. The last column shows the average ARI score of each method. (B) Bar plots showing the average ARI values on the 15 real scRNA-seq datasets using scMAE and seven comparative methods. (C) Bar plots showing the average NMI values on the 15 real scRNA-seq datasets using scMAE and seven comparative methods. (D) UMAP visualization of the cell embeddings for Macosko datasets learned by scMAE and comparative methods. The colors represent the clustering labels of each method. (E) UMAP visualization of the cell embeddings for Macosko datasets learned by scMAE and comparative methods. The colors represent the true cell types.

Based on the ARI values of each method on each dataset, a higher rank indicates a larger ARI value and better clustering performance for that method (Fig. 2A). scMAE achieved the highest ARI scores on 10 datasets, including Lung, Melanoma, Young, Guo, Baron, Spleen, Bach, Shekhar, Macosko, and Hrvatin. This indicates its robustness and accuracy in clustering cells from diverse platforms, tissues, and organisms. Furthermore, in nine datasets, scMAE outperformed the second-best method by a margin of >0.01 in terms of ARI. Notably, in datasets such as Braon, Shekhar, and Macosko, the ARI values of scMAE were >0.1 higher than the second-best method. In the remaining five datasets, scMAE’s ARI value was slightly lower than the highest ARI value, with a difference of <0.01 on two datasets (Limb_Muscle and Wang), and a difference of 0.02 on the Tosches, Worm_Neuron and Pollen datasets.

scMAE achieved the highest average rank value among the 15 datasets (Fig. 2A). The next best methods were scNAME, graph-sc, and contrastive-sc. It is worth noting that scNAME, contrastive-sc, and CLEAR are all based on contrastive learning with different augmentation methods. However, their performances varied significantly, highlighting the importance of augmentation in contrastive learning. The best-performing method among these three contrastive-based methods, scNAME, used the same augmentation method as scMAE, which involves generating a masked gene expression matrix. This demonstrates the effectiveness of this type of augmentation for deep learning models on scRNA-seq data. Overall, this comparison emphasizes the superior clustering performance of scMAE compared to existing methods, indicating its effectiveness in accurately identifying and characterizing cell clusters in single-cell RNA-seq data. As shown in Fig. 2B and C (see Supplementary Tables S2 and S3 for details), overall, scMAE exhibits superior performance by achieving a significantly higher average ARI and NMI score across all datasets, compared to other methods. In terms of cell-type ASW, we observed that contrastive-sc achieved the best performance, followed by scMAE (Supplementary Fig. S1 and Supplementary Table S4).

Moreover, to provide an intuitive understanding of the low-dimensional cell representations, we visualized the cell embeddings for each dataset (Fig. 2D and E, Supplementary Figs S2–S7). In this visualization, each point corresponds to a cell, and the cells are colored according to the clustering labels derived from each method as well as the actual labels. In the case of the Macosko dataset (Macosko *et al.* 2015), which primarily comprises rod cells, bipolar cells, amacrine cells, Müller glia, cone cells, and retinal ganglion cells, along with a few fibroblasts, microglia, pericytes, vascular or endothelium cells, astrocytes, and horizontal cells, as shown in Fig. 2D, only scMAE was able to clearly define the boundaries of rare cell type clusters, such as fibroblasts, microglia, and horizontal cells. Our results indicate that scMAE tends to distinguish different cell types effectively across 15 real scRNA-seq datasets. These results demonstrate that scMAE has learned cell representations that retain cell type information.

### 3.2 Ablation analysis

In this experiment, we conducted ablation experiments to analyze the effects of each component in the scMAE method. Three scenarios were considered: (i) removing the weighted reconstruction loss for unmasked data and only reconstructing the unmasked portion (referred to as scMAE-U); (ii) removing the weighted reconstruction loss for masked data and only reconstructing the masked portion (referred to as scMAE-M); (iii) removing the mask estimation loss (referred to as scMAE-W). The scatter plot in Supplementary Fig. S8A shows the ARI values for these three scenarios, as well as the scenario without any removal, using the scMAE method. The results indicate that reconstructing only the unmasked portion leads to worsened or similar performance for most datasets, with only slight improvement observed for the Baron dataset. On the other hand, reconstructing only the masked portion significantly reduces the clustering accuracy for most datasets, especially for the Shekhar, Spleen, Macosko, and Worm_Neuron datasets. For most datasets, the mask estimation loss positively impacts the clustering performance, except for the Pollen and Limb_Muscle datasets where the performance changes are minimal. Supplementary Figure S8B presents the average ARI, NMI, and silhouette coefficient values (Rousseeuw 1987) for the 15 datasets under the three ablation experiments and the scMAE method. The results indicate that both the weighted reconstruction loss and the mask estimation loss contribute to improved clustering performance, highlighting the effectiveness of all the loss functions in scMAE. Furthermore, to visually demonstrate the effectiveness of the scMAE loss functions, we visualize the cell embeddings of the Macosko dataset (Macosko *et al.* 2015) under scMAE and the three ablation scenarios (Supplementary Fig. S8C). Compared to the original scMAE, the clusters in the ablation scenarios appear more scattered, especially when reconstructing only the masked data, where the clusters are mixed.

To evaluate the impact of the masked input data ratio, the weighting of the masked portion in the reconstruction loss, the weighting parameters of the two objective functions, and the learning rate on the clustering results, we conducted a comprehensive sensitivity analysis of hyperparameters. The results demonstrate the robustness of the chosen masked data ratio within the range of 0.2–0.4. The default values for the weights of the masked portion in the reconstruction loss, weights for the two objective functions, and the learning rate were determined based on extensive hyperparameter search experiments, as summarized in Supplementary Fig. S9 and Supplementary Note S2.

### 3.3 scMAE can accurately identify rare cell types

To demonstrate the accurate identification of rare cell types by scMAE, we conducted a detailed analysis of the Shekhar dataset. This dataset consists of mouse retinal bipolar cells, with a total of 18 cell subtypes, including bipolar cells (BC), Rod Bipolar cells (RBC), Müller glia (MG), amacrine cells (AC), and photoreceptors (PR) (Shekhar *et al.* 2016). The BC cells further divide into 13 subpopulations (BC1A, BC1B, BC2, BC3A, BC3B, BC4, BC5A, BC5B, BC5C, BC5D, BC6, BC7, and BC8/9), and the PR cells are categorized as Rod Photoreceptors (Rod PC) and Cone Photoreceptors (Cone PC). It is worth noting that the Rod PR and Cone PR cells are extremely rare, accounting for only 0.003% and 0.001% of the total cell population, respectively.

**Figure 3.**
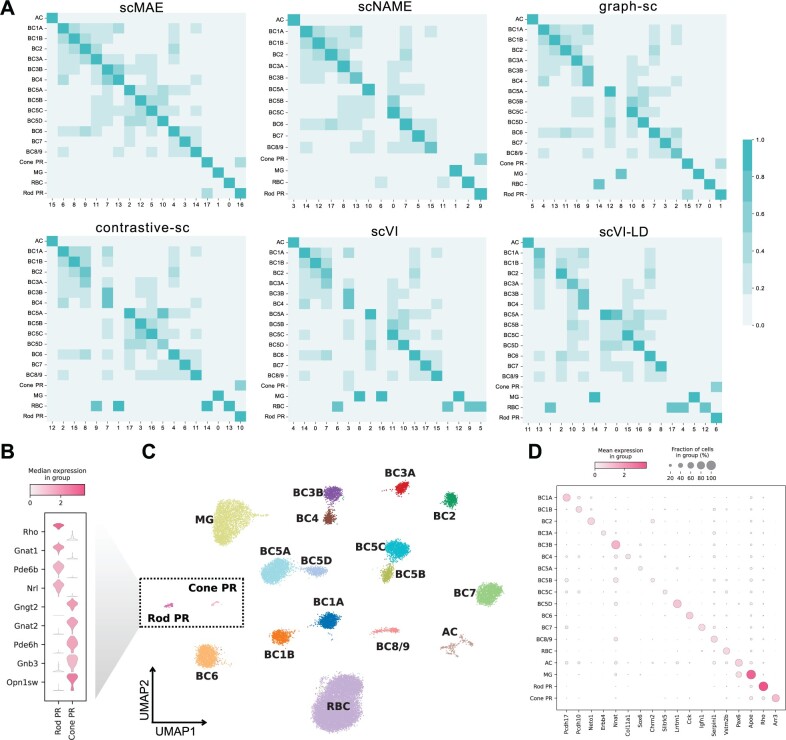
scMAE can accurately identify rare cell types. (**A**) Overlap of top 50 differentially expressed genes in clusters detected by scMAE and comparative methods with true cell types. (B) Violin plot showing the differential expression genes of the Rod PR cluster and the Cone PR cluster. (C) UMAP visualization of the cell embeddings for the Macosko dataset learned by scMAE. The colors represent the scMAE clustering labels. (D) Dot plot showing the marker genes of the clusters.

Following scNAME (Wan *et al.* 2022), we performed differential expression analysis using the true labels, scMAE clustering labels, and labels obtained from the comparative methods to identify differentially expressed genes (DEGs) in each cluster. To validate the superiority of scMAE, we compared the overlap between the top 50 DEGs in each cluster identified by scMAE and the seven comparative methods and the DEGs in the true cell types. In Fig. 3A and Supplementary Fig. S10, each row represents a cluster obtained by a specific method, and each column represents a known cell type. The color depth indicates the degree of overlap between the DEGs from the true labels and the DEGs from the clustering labels. scMAE achieved the highest overlap with the true labels using the top 50 most important DEGs, allowing for the assignment of a unique cluster to each cell type. However, scNAME failed to accurately identify BC5B and Cone PR cells, graph-sc could not assign a cell type to Cluster 9, RBC cells were distributed across Clusters 14 and 0, and MG cells were found in Clusters 8 and 17. contrastive-sc identified Clusters 9, 1, 14, and 13 as RBC cells, and Cluster 7 contained both BC3B and BC4 subpopulations, but no match was found for the Cone PR cell type. Both scVI and scVI-LD identified BC3B and BC4 as the same cluster. Moreover, scVI failed to recognize two rare cell types, Cone PR and Rod PR, while scVI-LD grouped these two rare types into the same cluster. scGNN incorrectly estimated the number of clusters, and CLEAR failed to accurately identify Cone PR and Rod PR cells. In other words, only scMAE was able to annotate each cluster to a known cell type.

Based on these results, we annotated the clustering results of scMAE (Fig. 3C). In addition, we performed separate differential gene expression analyses for Rod PR and Cone PR cells, revealing that Rod PR cells highly expressed genes such as Rho and Gnat1, while Cone PR cells exhibited high expression of Gngt2, Gnat2, Pde6h, Gnb3, and Opn1sw genes (Fig. 3B). Rho and Gnat1 are primarily associated with rod cells and visual signal transduction (Botta *et al.* 2016), while Gngt2, Gnat2, Pde6h, Gnb3, and Opn1sw genes are collectively involved in visual signal transduction processes in cone cells (Mustafi *et al.* 2011). We also presented bubble plots of marker genes defining the cell types as described in the literature (Shekhar *et al.* 2016) (Fig. 3D), further validating the ability of scMAE to accurately distinguish subtypes of mouse retinal bipolar cells.

### 3.4 Biological analysis of scMAE clustering results

We have demonstrated the outperforming clustering results provided by scMAE based on evaluation metrics. However, it is also important to interpret these results biologically in practical applications. Therefore, we further explore the biological implications of the clustering results using the Hrvatin dataset (Hrvatin *et al.* 2018). This dataset consists of adult mouse visual cortex cells and includes eight major cell types: excitatory neurons, inhibitory neurons, oligodendrocytes, oligodendrocyte precursor cells (OPCs), astrocytes, endothelial and smooth muscle cells, pericytes, microglia, and macrophages (Hrvatin *et al.* 2018).

**Figure 4.**
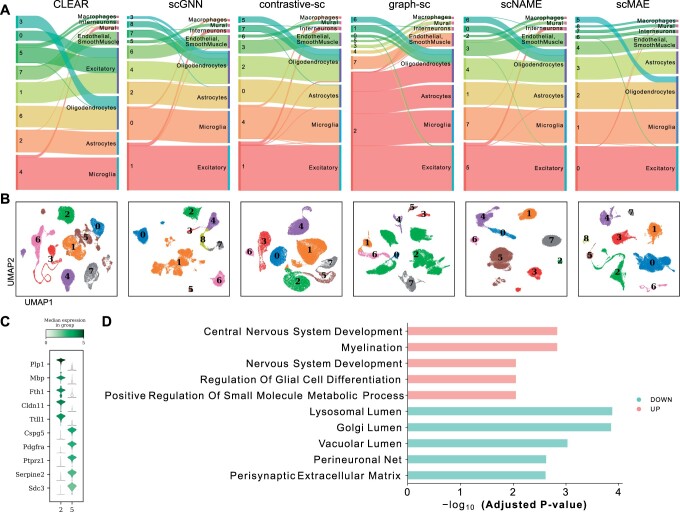
Biological analysis in the Hrvatin dataset. (A) Sankey plots of clustering results and true cell types for scMAE and comparative methods. For each subplot, the left side represents the clustering labels generated by each method, while the right side represents the true cell types. (B) UMAP visualization of the cell embeddings learned by scMAE and comparative methods. The colors represent the clustering labels assigned by each method. (C) Violin plot showing the differential expression genes of Cluster 2 and Cluster 5. (D) The enriched Gene Ontology (GO) terms in Cluster 2 versus Cluster 5.

To visually assess the accuracy of each method’s clustering, Fig. 4A and Supplementary Fig. S11 display the clustering labels on the left side of each plot and the true cell labels on the right side. The thickness of the connecting lines represents the number of matching labels between the clustering and the ground truth. scMAE effectively distinguishes all cell types and separates oligodendrocytes into two clusters. However, scNAME merges interneurons into excitatory neurons and mural into endothelial and smooth muscle cells. graph-sc successfully distinguishes interneurons and mural, but Cluster 2 contains astrocytes, microglia, excitatory neurons, and oligodendrocyte precursor cells. contrastive-sc fails to identify macrophages and interneurons, while CLEAR fails to recognize macrophages and interneurons. In the clustering by scVI, Macrophages cells and Excitatory cells were mixed, and interneurons and Oligodendrocytes were also combined. Similarly, scVI-LD did not accurately identify Macrophages and interneurons as distinct cell types.

Figure 4B presents the UMAP embeddings of each method, where different colors represent the clustering labels. Moreover, we conducted differential gene expression analysis and performed functional annotation to characterize the distinct functions of Cluster 2 and Cluster 5, both labeled as oligodendrocytes. The violin plots in Fig. 4C depict the expression profiles of the top five differentially expressed genes. Notably, Pdgfra and Ptprz1 are known marker genes for oligodendrocyte precursors (Marques *et al.* 2016). Enrichment analysis reveals that Cluster 2 is associated with Myelination and neural system development, while Cluster 5 is enriched in Golgi lumen and perisynaptic extracellular matrix-related signaling pathways (Fang *et al.* 2023) (Fig. 4D). These findings provide valuable insights into the biological interpretation of the clustering results obtained by scMAE, highlighting specific cell types and their functional characteristics within the adult mouse visual cortex.

## 4 Discussion

In this study, we have developed a novel single-cell denoising autoencoder model called scMAE for identifying cell types in scRNA-seq datasets. Unlike previous autoencoder models, scMAE introduces partial corruption to the gene expression data and incorporates a masking predictor to capture the correlations between genes. Specifically, scMAE takes the corrupted data as input to the encoder, obtains a low-dimensional embedding, and then passes it to the masking predictor. The masking predictor predicts whether a feature is corrupted by comparing its value with similar features. The predicted results and the embedding features are then fed into the decoder to reconstruct the original gene expression values.

The encoder’s ability to capture feature correlations and generate informative embeddings proved to be critical in achieving effective reconstruction. The mask predictor further contributed to the model’s performance by identifying masked features based on gene value inconsistencies. In addition, the decoder’s prior knowledge of corrupted features was instrumental in accurately filling in missing information, leading to meaningful cell representation. We visualized the loss during the training process for 15 datasets and observed a continuous decrease in loss as training progressed, especially the rapid convergence of Mask estimation loss to near 0 in the initial epochs (Supplementary Fig. S12). This also indicates that the Mask predictor accurately predicts which expression values are corrupted, which is crucial for the successful reconstruction by the decoder.

Our experimental results demonstrate that scMAE achieves excellent clustering performance on 15 datasets, outperforming seven state-of-the-art clustering methods designed for scRNA-seq data. These methods are based on contrastive learning and graph neural networks, and they have been proven to outperform previous autoencoder-based methods. In particular, scMAE excelled in accurately identifying rare cell types, showcasing its potential for discovering subtle cellular differences. Therefore, we believe that appropriate corruption or perturbation of gene expression data facilitates the learning of higher-order features by the encoder. scMAE shows efficient runtime and exhibits memory performance comparable to most methods (Supplementary Fig. S13). Furthermore, we conducted experiments on two datasets with batch effects and found that scMAE achieves satisfactory clustering results when handling data with batch effects. (Supplementary Fig. S14) and (Supplementary Note S3).

As high-throughput scRNA-seq technologies and cell atlases continue to evolve, we will explore the performance of scMAE on larger-scale scRNA-seq datasets in the future. In addition, considering the integration of annotated cell information to achieve more accurate identification of cell subtypes (Qiu *et al.* 2023). The effectiveness of scMAE in accurately identifying and grouping cells based on gene expression profiles contributes to a deeper understanding of cellular heterogeneity and functional diversity.

## Supplementary Material

btae020_Supplementary_Data
