# Supplementary material for: scMAE: a masked autoencoder for single-cell RNA-seq clustering
Source: Bioinformatics. 2024 Jan 16;40(1):btae020. doi: 10.1093/bioinformatics/btae020 (PMC10832357; doi:10.1093/bioinformatics/btae020)
Supplement: btae020_Supplementary_Data [file btae020_supplementary_data.docx]

**Supplementary Materials For**

**scMAE: a masked autoencoder for single-cell RNA-seq clustering**

**Zhao-Yu Fang^1^, Ruiqing Zheng^1^, Min Li^1*^**

^1^School of Computer Science and Engineering, Central South University, Changsha, Hunan 410083, P.R. China

*To whom correspondence should be addressed. Email: limin@mail.csu.edu.cn.

**Supplementary Tables**

**Supplementary Table 1.** Summary of the real single-cell RNA-seq datasets.

**Supplementary Table 2.** ARI values of scMAE and 7 competitive methods in 15 scRNA-seq datasets.

**Supplementary Table 3.** NMI values of scMAE and 7 competitive methods in 15 scRNA-seq datasets.

**Supplementary Table 4.** Cell-type ASW values of scMAE and 7 competitive methods in 15 scRNA-seq datasets

**Supplementary Figures**

**Supplementary Figure 1**. Bar plots showing the average Cell-type ASW values on the 15 real datasets using scMAE and 7 comparison methods.

**Supplementary Figure 2-Figure 5.** UMAP-visualization of the cell embeddings learned by scMAE and 5 competitive methods (scGNN, CLEAR, contrastive-sc, graph-sc, CLEAR).

**Supplementary Figure 6-Figure 7.** UMAP-visualization of the cell embeddings learned by scVI and scVI-LD.

**Supplementary Figure 8.** Ablation study for scMAE.

**Supplementary Figure 9.** Identification of optimal parameters.

**Supplementary Figure 10.** Overlap of top 50 differentially expressed genes in clusters detected by scGNN and CLEAR with true cell types.

**Supplementary Figure 11.** Sankey plots of clustering results and true cell types for scVI and scVI-LD.

**Supplementary Figure 12.** The loss value over iteration on the 15 datasets.

**Supplementary Figure 13.** Running time and memory consumption for scMAE and 7 comparison methods.

**Supplementary Figure 14.** Clustering results on MouseRetian dataset for scMAE and 6 comparison methods.

**Supplementary Notes**

**Supplementary Note 1.** Details in data-preprocessing.

**Supplementary Note 2.** Hyperparameter configuration of scMAE.

**Supplementary Note 3.** Clustering results of data with batch effects.

**Supplementary Table 1.** Summary of the real single-cell RNA-seq datasets

| Dataset | Description | n cells | n genes | n clusters | Platform | Reference |
| --- | --- | --- | --- | --- | --- | --- |
| Pollen | Cerebral cortex | 301 | 21721 | 11 | unknown | [1] |
| Lung | Mouse lung | 1676 | 23341 | 11 | Smart-seq2 | [2] |
| Limb_Muscle | Mouse limb muscle | 3909 | 23341 | 6 | 10x | [2] |
| Worm_Neuron | Worm neuron cells | 4186 | 13488 | 10 | sci-RNA-seq | [3] |
| Melanoma | Metastatic melanoma | 4513 | 23684 | 9 | Smart-seq2 | [4] |
| Young | Human kidneys | 5685 | 33658 | 11 | unknown | [5] |
| Guo | Adult human testis | 6490 | 27477 | 12 | 10x | [6] |
| Baron | Human Pancreas | 8569 | 20125 | 14 | inDrop | [7] |
| Wang | Pulmonary alveolar type I cell | 9519 | 14561 | 2 | 10x | [8] |
| Spleen | Mouse spleen | 9552 | 23341 | 5 | 10x | [2] |
| Tosches | Reptilian brain | 18664 | 23500 | 15 | Drop-seq | [9] |
| Bach | Mammary epithelial cells | 23184 | 19965 | 8 | 10x | [10] |
| Shekhar | Mouse retinal bipolar cells | 26830 | 13166 | 18 | Drop-seq | [11] |
| Macosko | Mouse retinal cells | 44808 | 23288 | 12 | Drop-seq | [12] |
| Hrvatin  MouseRetina | Mouse visual cortex  Mouse retina | 48266  71638 | 25187  12333 | 8  12 | Drop-seq  Drop-seq | [13]  [14] |

**Supplementary Table 2.** ARI values of scMAE and 7 competitive methods in 15 scRNA-seq datasets

| datasets | CLEAR | scGNN | contrastive-sc | scVI | graph-sc | scVI-LD | scNAME | scMAE |
| --- | --- | --- | --- | --- | --- | --- | --- | --- |
| Pollen | 0.151 | 0.705 | 0.831 | **0.952** | 0.911 | 0.875 | 0.924 | 0.936 |
| Lung | 0.102 | 0.387 | 0.600 | 0.423 | 0.743 | 0.522 | 0.643 | **0.749** |
| Limb_Muscle | 0.809 | 0.583 | **0.989** | 0.942 | 0.978 | 0.986 | 0.982 | 0.983 |
| worm_neuron | 0.188 | 0.061 | 0.155 | 0.481 | 0.477 | **0.511** | 0.251 | 0.487 |
| Melanoma | 0.240 | 0.467 | 0.519 | 0.527 | 0.654 | 0.503 | 0.639 | **0.750** |
| Young | 0.452 | 0.237 | 0.603 | 0.715 | 0.664 | 0.470 | 0.629 | **0.770** |
| Guo | 0.638 | 0.424 | 0.812 | 0.603 | 0.736 | 0.733 | 0.814 | **0.839** |
| Baron | 0.466 | 0.384 | 0.495 | 0.541 | 0.643 | 0.526 | 0.657 | **0.771** |
| Wang | 0.400 | 0.235 | 0.937 | 0.927 | -0.039 | 0.742 | **0.943** | 0.936 |
| Spleen | 0.414 | 0.379 | 0.906 | 0.323 | 0.905 | 0.508 | 0.918 | **0.928** |
| Tosches | 0.494 | 0.512 | 0.392 | 0.566 | **0.751** | 0.645 | 0.584 | 0.726 |
| Bach | 0.609 | 0.672 | 0.816 | 0.741 | 0.809 | 0.828 | 0.850 | **0.890** |
| Shekhar | 0.412 | 0.448 | 0.463 | 0.467 | 0.675 | 0.558 | 0.712 | **0.979** |
| Macosko | 0.270 | 0.372 | 0.194 | 0.332 | 0.395 | 0.257 | 0.805 | **0.920** |
| Hrvatin | 0.669 | 0.733 | 0.861 | 0.751 | 0.168 | 0.889 | 0.873 | **0.931** |
| Average | 0.421 | 0.440 | 0.638 | 0.619 | 0.631 | 0.637 | 0.748 | **0.840** |

Note: Highest ARI value for each dataset is in bold. The last row shows the average ARI score of each method.

**Supplementary Table 3.** NMI values of scMAE and 7 competitive methods in 15 scRNA-seq datasets

| datasets | CLEAR | scGNN | contrastive-sc | scVI | graph-sc | scVI-LD | scNAME | scMAE |
| --- | --- | --- | --- | --- | --- | --- | --- | --- |
| Pollen | 0.306 | 0.808 | **0.947** | 0.916 | 0.894 | 0.922 | 0.923 | 0.930 |
| Lung | 0.286 | 0.642 | 0.705 | 0.726 | 0.765 | 0.820 | 0.796 | **0.824** |
| Limb_Muscle | 0.854 | 0.734 | 0.926 | 0.970 | **0.976** | 0.955 | 0.962 | 0.974 |
| worm_neuron | 0.325 | 0.211 | 0.635 | 0.673 | 0.308 | **0.697** | 0.456 | 0.684 |
| Melanoma | 0.385 | 0.606 | 0.631 | 0.615 | 0.618 | 0.731 | 0.713 | **0.745** |
| Young | 0.576 | 0.375 | 0.777 | 0.649 | 0.730 | 0.778 | 0.771 | **0.824** |
| Guo | 0.795 | 0.628 | 0.796 | 0.827 | 0.839 | 0.818 | 0.838 | **0.859** |
| Baron | 0.723 | 0.572 | 0.777 | 0.768 | 0.674 | 0.807 | 0.809 | **0.819** |
| Wang | 0.405 | 0.438 | 0.862 | 0.663 | **0.867** | 0.020 | 0.878 | 0.864 |
| Spleen | 0.534 | 0.501 | 0.557 | 0.638 | 0.802 | 0.843 | 0.846 | **0.861** |
| Tosches | 0.752 | 0.500 | 0.775 | **0.810** | 0.654 | 0.774 | 0.775 | 0.799 |
| Bach | 0.753 | 0.764 | 0.788 | 0.811 | 0.800 | 0.818 | **0.854** | 0.852 |
| Shekhar | 0.790 | 0.516 | 0.800 | 0.818 | 0.791 | 0.851 | 0.856 | **0.952** |
| Macosko | 0.546 | 0.414 | 0.599 | 0.569 | 0.518 | 0.594 | 0.718 | **0.851** |
| Hrvatin | 0.812 | 0.855 | 0.846 | 0.899 | 0.895 | 0.491 | 0.909 | **0.959** |
| Average | 0.589 | 0.571 | 0.762 | 0.757 | 0.742 | 0.728 | 0.807 | **0.853** |

Note: Highest NMI value for each dataset is in bold. The last row shows average NMI score of each method.

**Supplementary Table 4.** Cell-type ASW values of scMAE and 7 competitive methods in 15 scRNA-seq datasets

| datasets | CLEAR | scGNN | contrastive-sc | scVI | graph-sc | scVI-LD | scNAME | scMAE |
| --- | --- | --- | --- | --- | --- | --- | --- | --- |
| Pollen | 0.505 | 0.663 | 0.786 | 0.719 | 0.736 | 0.777 | 0.682 | **0.791** |
| Lung | 0.498 | 0.579 | **0.750** | 0.594 | 0.748 | 0.679 | 0.652 | 0.736 |
| Limb_Muscle | 0.571 | 0.608 | **0.809** | 0.614 | 0.779 | 0.685 | 0.639 | 0.711 |
| worm_neuron | 0.508 | 0.480 | 0.484 | 0.539 | 0.571 | **0.577** | 0.497 | 0.557 |
| Melanoma | 0.506 | 0.566 | 0.598 | 0.567 | **0.632** | 0.585 | 0.546 | 0.601 |
| Young | 0.531 | 0.525 | 0.664 | 0.596 | **0.665** | 0.613 | 0.560 | 0.644 |
| Guo | 0.573 | 0.617 | **0.773** | 0.636 | 0.735 | 0.702 | 0.630 | 0.736 |
| Baron | 0.576 | 0.523 | 0.647 | 0.601 | **0.685** | 0.639 | 0.584 | 0.676 |
| Wang | 0.511 | 0.711 | **0.842** | 0.581 | 0.717 | 0.618 | 0.733 | 0.750 |
| Spleen | 0.544 | 0.594 | **0.834** | 0.599 | 0.774 | 0.627 | 0.665 | 0.764 |
| Tosches | 0.574 | 0.523 | **0.686** | 0.625 | 0.637 | 0.668 | 0.616 | 0.645 |
| Bach | 0.555 | 0.587 | **0.747** | 0.602 | 0.643 | 0.655 | 0.626 | 0.681 |
| Shekhar | 0.628 | 0.567 | **0.747** | 0.618 | 0.654 | 0.673 | 0.587 | 0.635 |
| Macosko | 0.550 | 0.598 | **0.646** | 0.617 | 0.635 | 0.626 | 0.538 | 0.628 |
| Hrvatin | 0.564 | 0.618 | **0.819** | 0.640 | 0.500 | 0.714 | 0.666 | 0.678 |
| Average | 0.546 | 0.584 | **0.722** | 0.610 | 0.674 | 0.656 | 0.615 | 0.682 |

Note: Highest Cell-type ASW value for each dataset is in bold. The last row shows average Cell-type ASW score of each method.


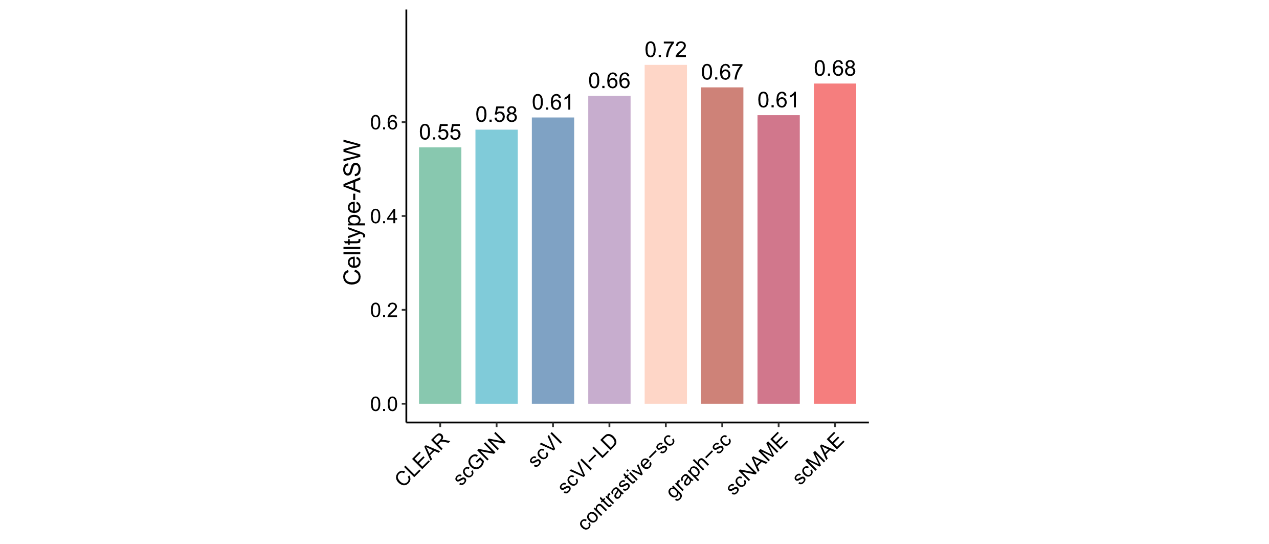


**Supplementary Figure 1.** Bar plots showing the average Cell-type ASW values on the 15 real dataset using scMAE and 7 comparsion methods.

**
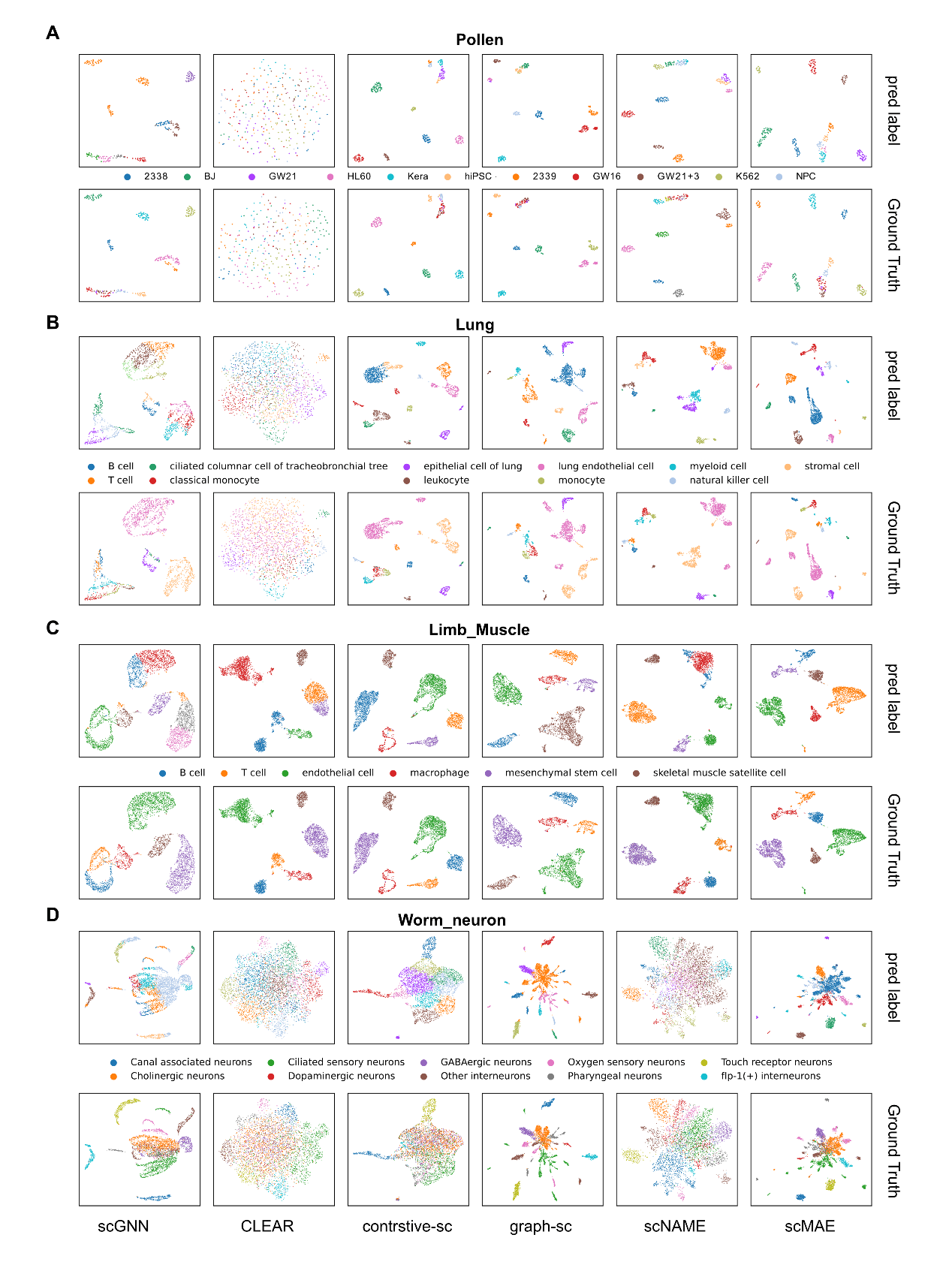
**

**Supplementary Figure 2.** UMAP-visualization of the cell embeddings for four scRNA-seq datasets, Pollen, Lung, Limb_Muscle, and Worm_neuron, learned by scMAE and five competitive methods. For each panel, the colors on the upper graphs represent the clustering labels of each method, while the colors on the lower graphs represent the true cell types.


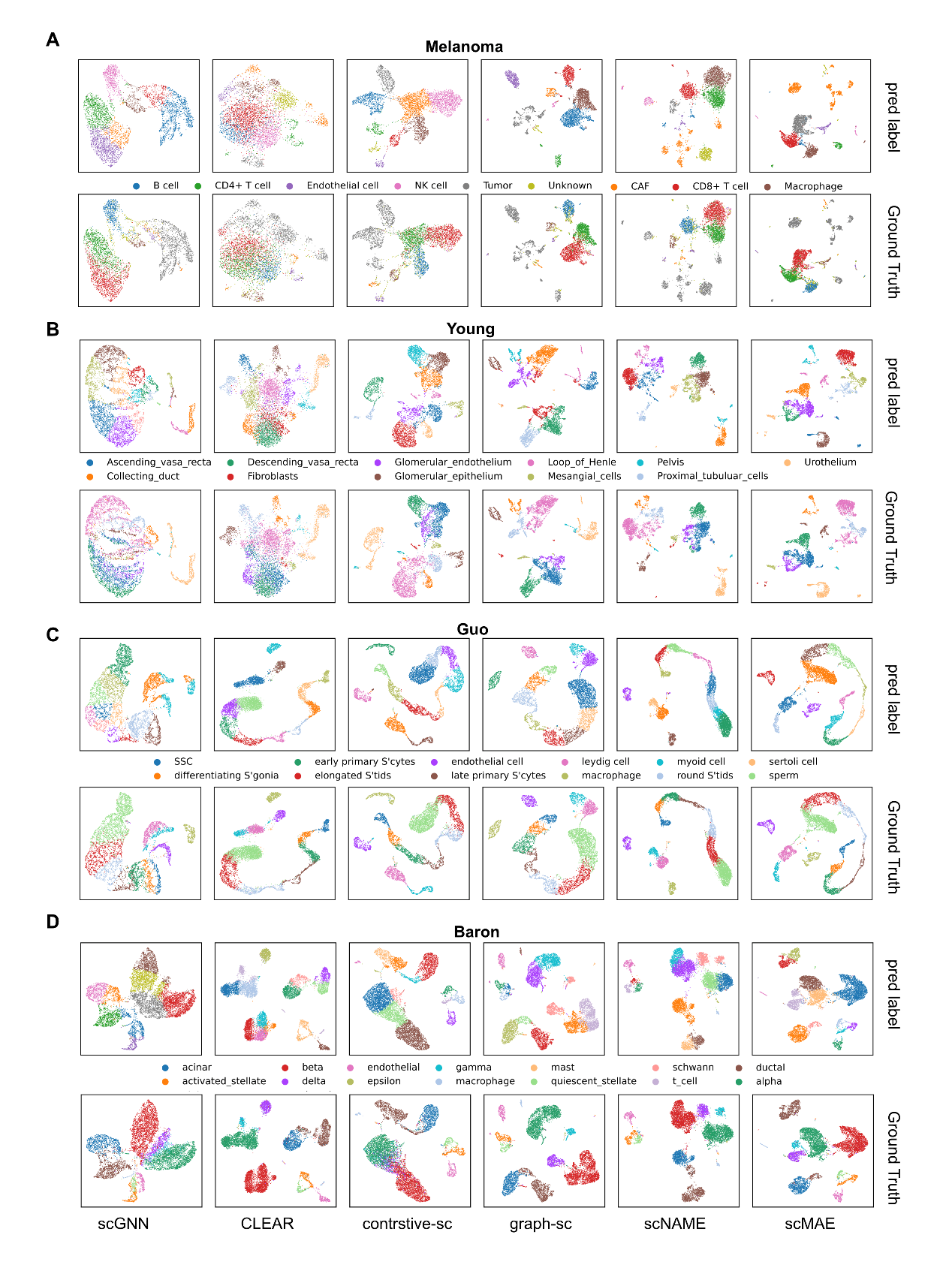


**Supplementary Figure 3.** UMAP-visualization of the cell embeddings for four scRNA-seq datasets, Melanoma, Young, Guo, and Baron, learned by scMAE and five competitive methods. For each panel, the colors on the upper graphs represent the clustering labels of each method, while the colors on the lower graphs represent the true cell types.


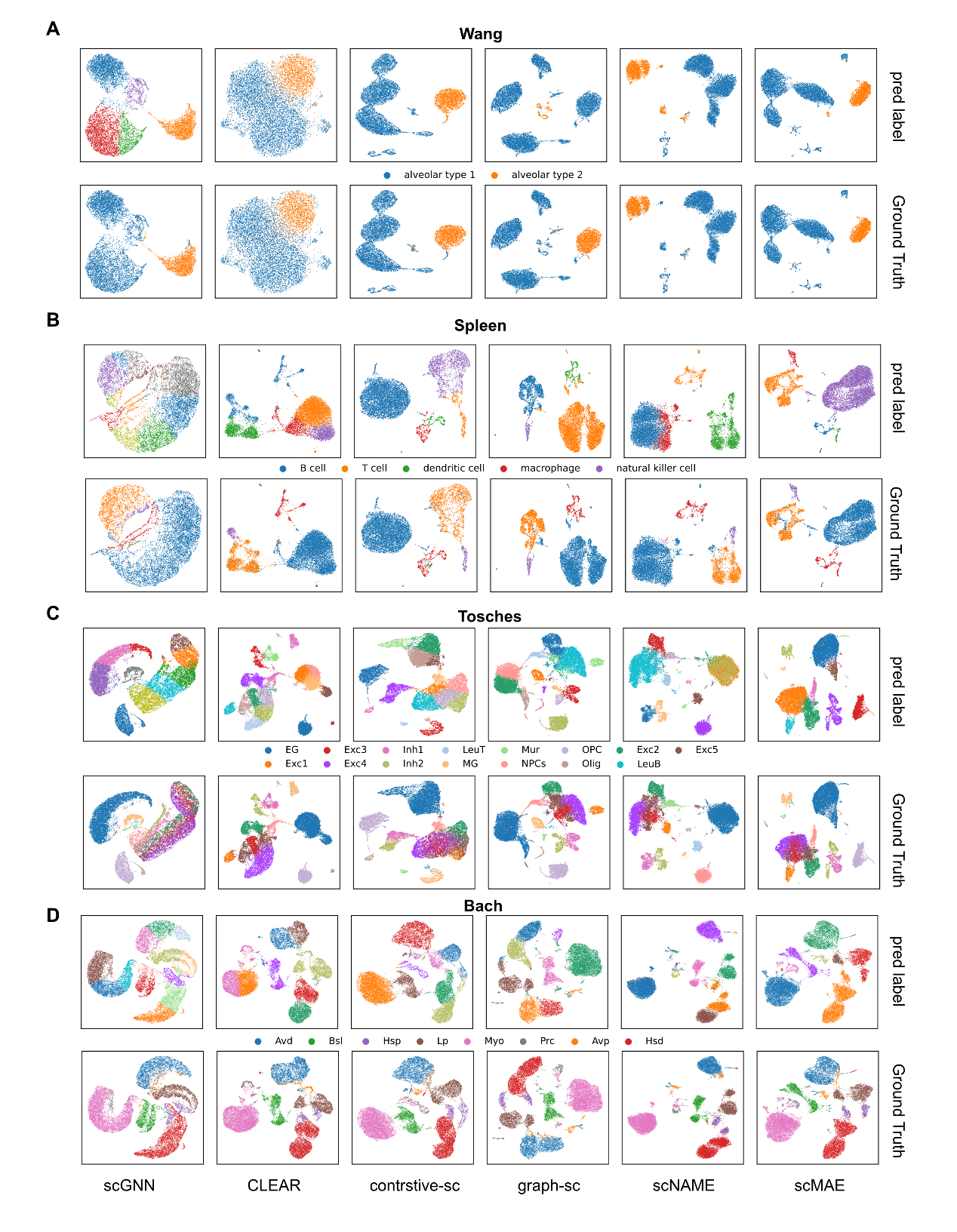


**Supplementary Figure 4.**UMAP-visualization of the cell embeddings for four scRNA-seq datasets, Wang, Spleen, Tosches, and Bach, learned by scMAE and five competitive methods. For each panel, the colors on the upper graphs represent the clustering labels of each method, while the colors on the lower graphs represent the true cell types.


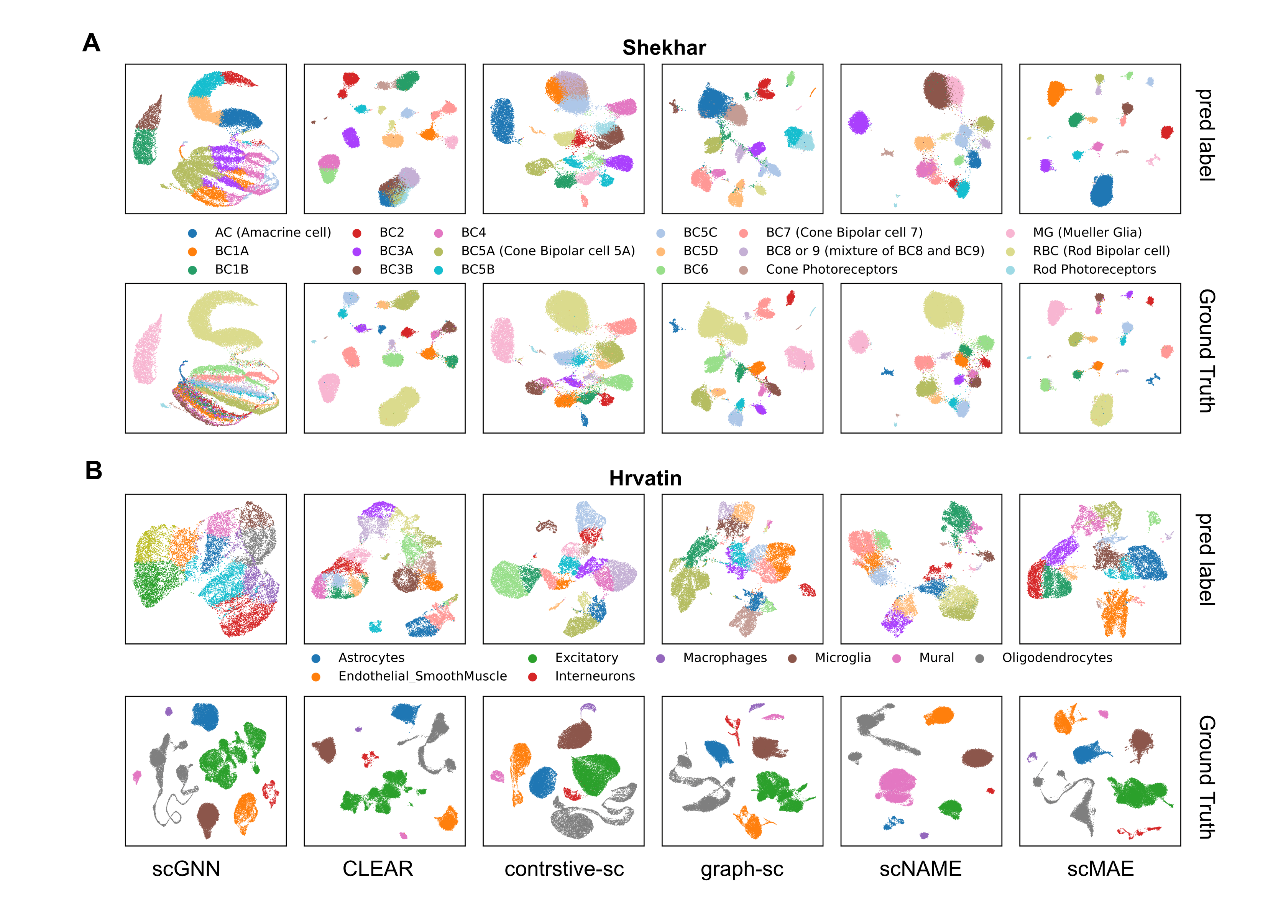


**Supplementary Figure 5.** UMAP-visualization of the cell embeddings for two scRNA-seq datasets, Shekhar, and Hrvatin, learned by scMAE and five competitive methods. For each panel, the colors on the upper graphs represent the clustering labels of each method, while the colors on the lower graphs represent the true cell types.


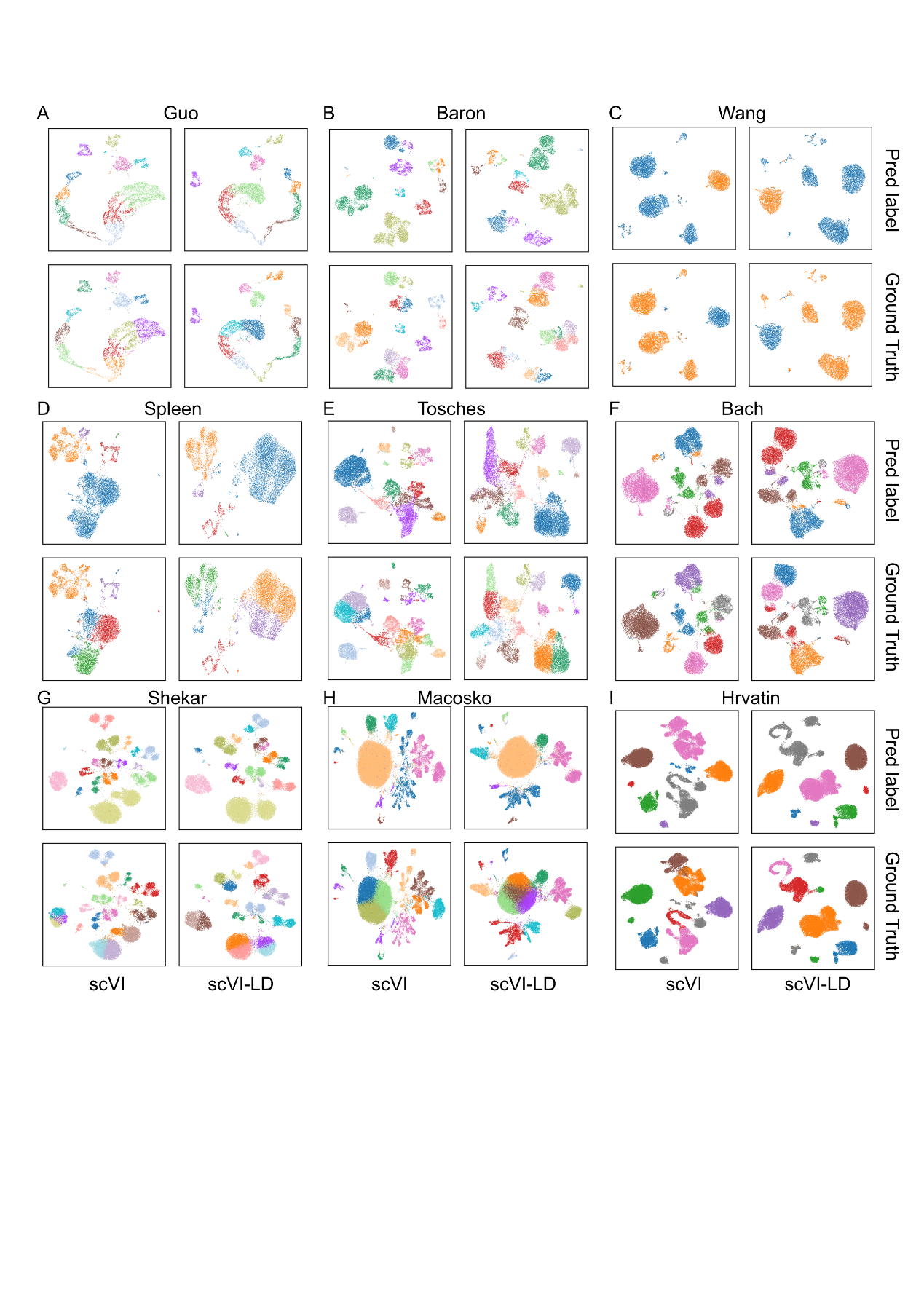


**Supplementary Figure 6**. UMAP visualization of the cell embeddings for Guo, Baron, Wang, Spleen, Tosches, Bach, Shekar, Macosko and Hrvatin datasets learned by scVI and scVI-LD.


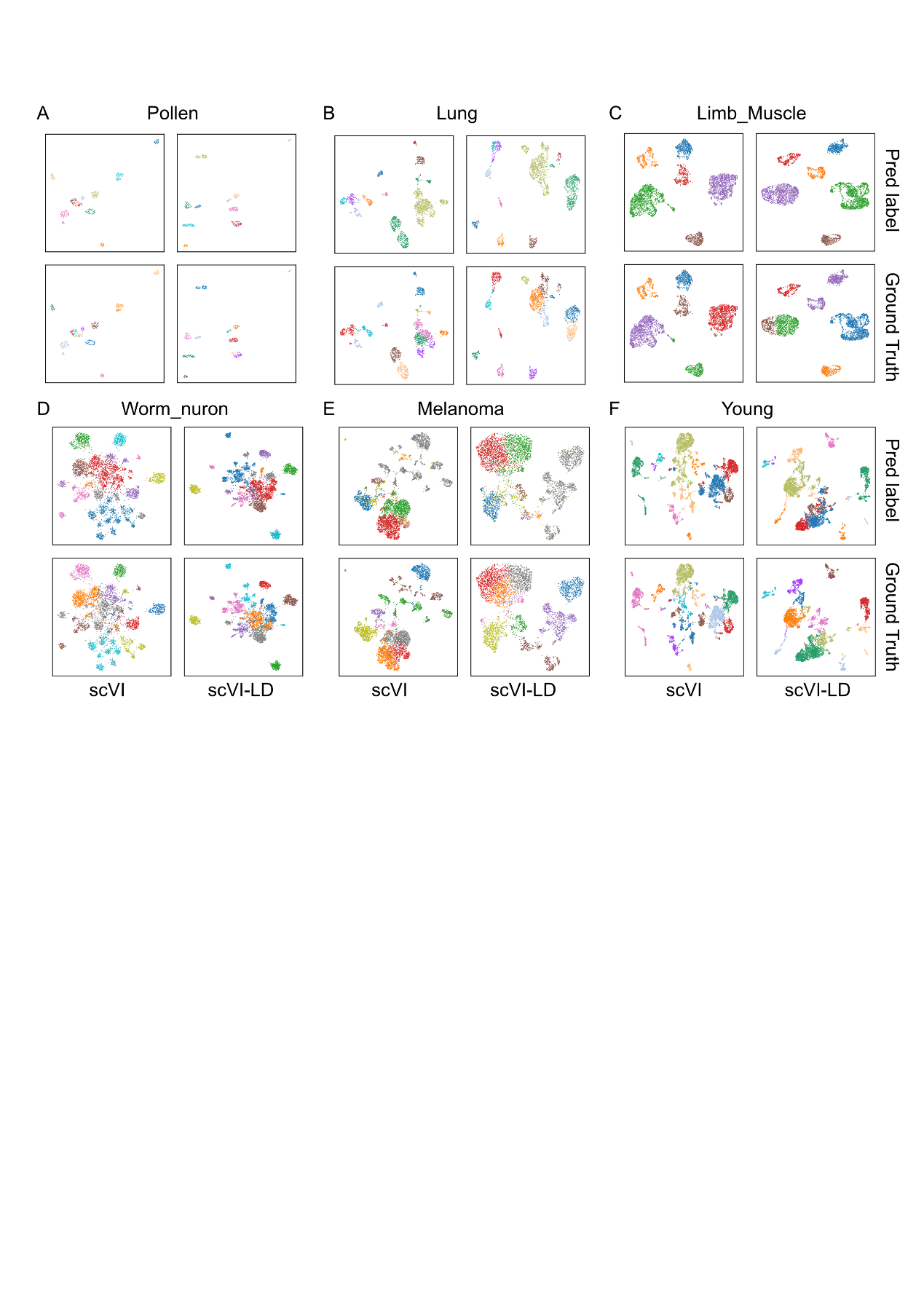


**Supplementary Figure 7.** UMAP visualization of the cell embeddings for Pollen, Lung, Limb_Muscle, Worm_nuron, Melanoma and Young datasets learned by scVI and scVI-LD methods.

**
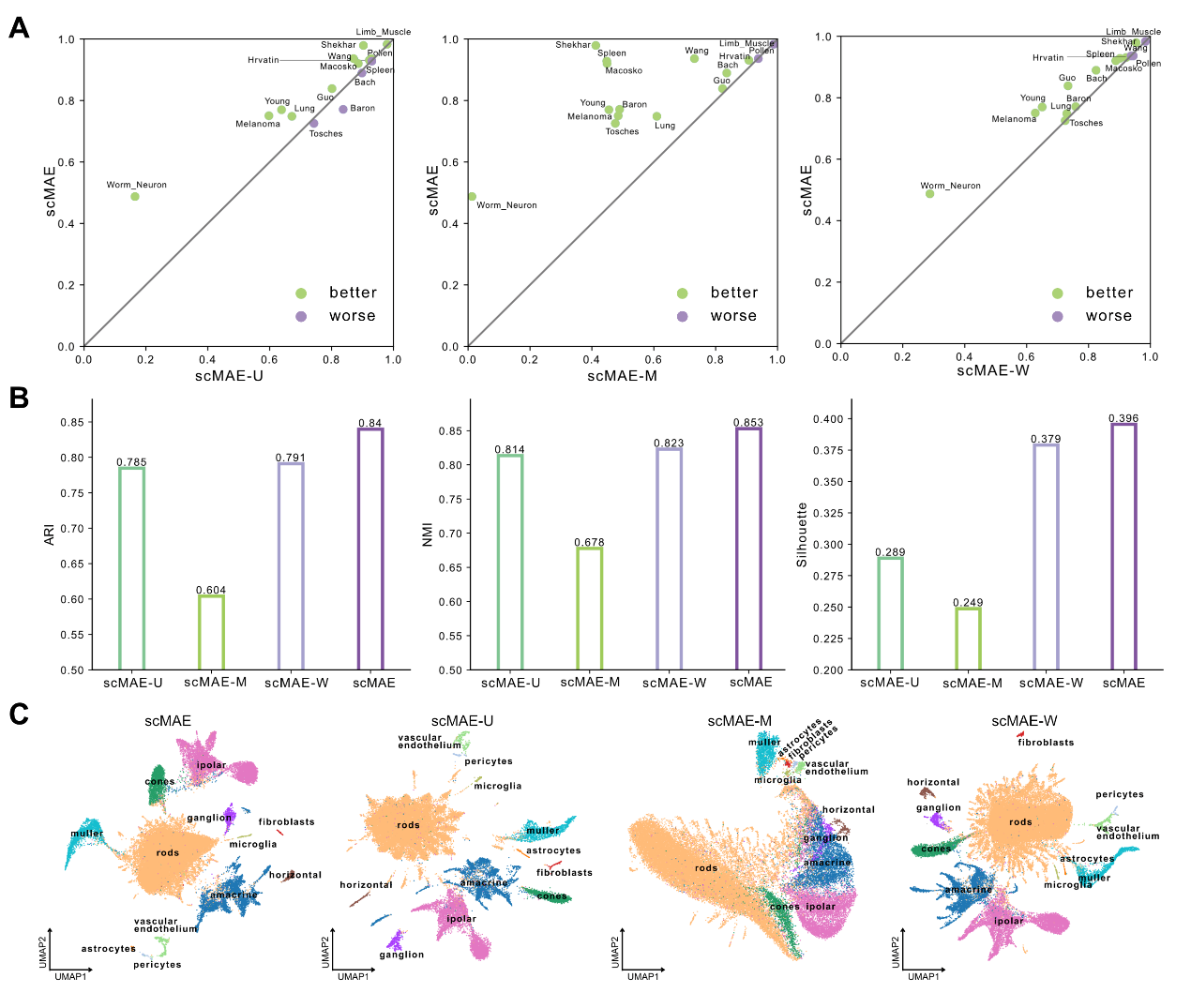
**

**Supplementary Figure 8.**Ablation study for scMAE. **A)** Comparison of ARI values for scMAE with respect to reconstructing only the unmasked data (scMAE-U), reconstructing only the masked data (scMAE-M), and without the mask estimation loss (scMAE-W). Purple dots indicate better performance for scMAE, while green dots indicate the opposite. **B)** Bar plots showing the average ARI values, NMI values, and silhouette coefficients for scMAE and 3 ablation methods on 15 real scRNA-seq datasets. **C)** UMAP visualization of the cell embeddings for the Macosko dataset learned by scMAE and the 3 ablation methods. The colors represent the true cell types.

**
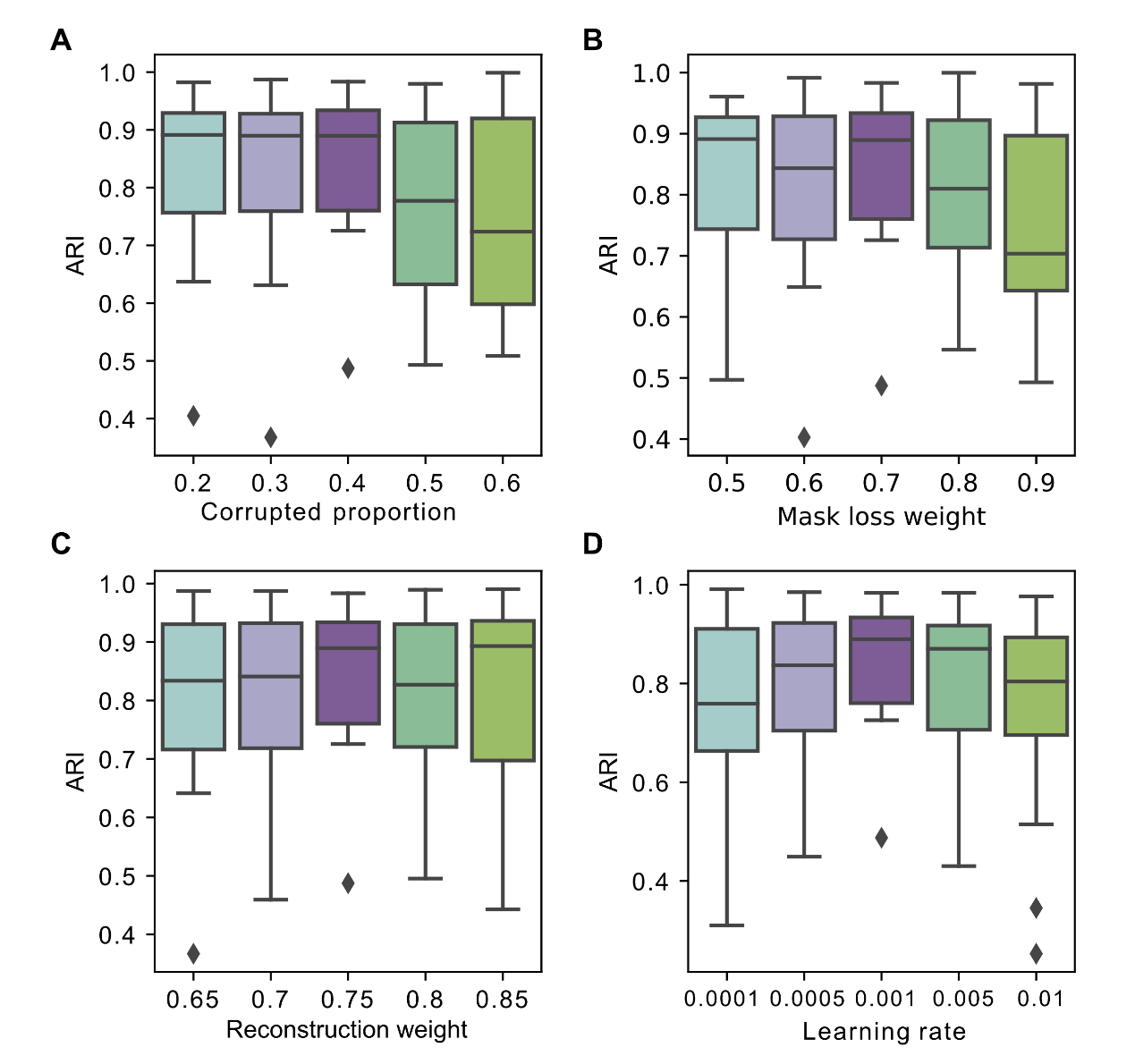
**

**Supplementary Figure 9.** Identification of optimal parameters. **A)** Illustrates the impact of different proportions of gene expression values being corrupted, ranging from 0.2 to 0.6. **B)** Examines the effect of varying the weight assigned to the masking estimation loss in the loss function, ranging from 0.5 to 0.9. **C)** Demonstrates the influence of adjusting the weight assigned to the corrupted gene expression values in the reconstruction loss, ranging from 0.65 to 0.85. **D)** Explores the effect of different learning rates, ranging from 1e-4 to 1e-2, on the overall performance.


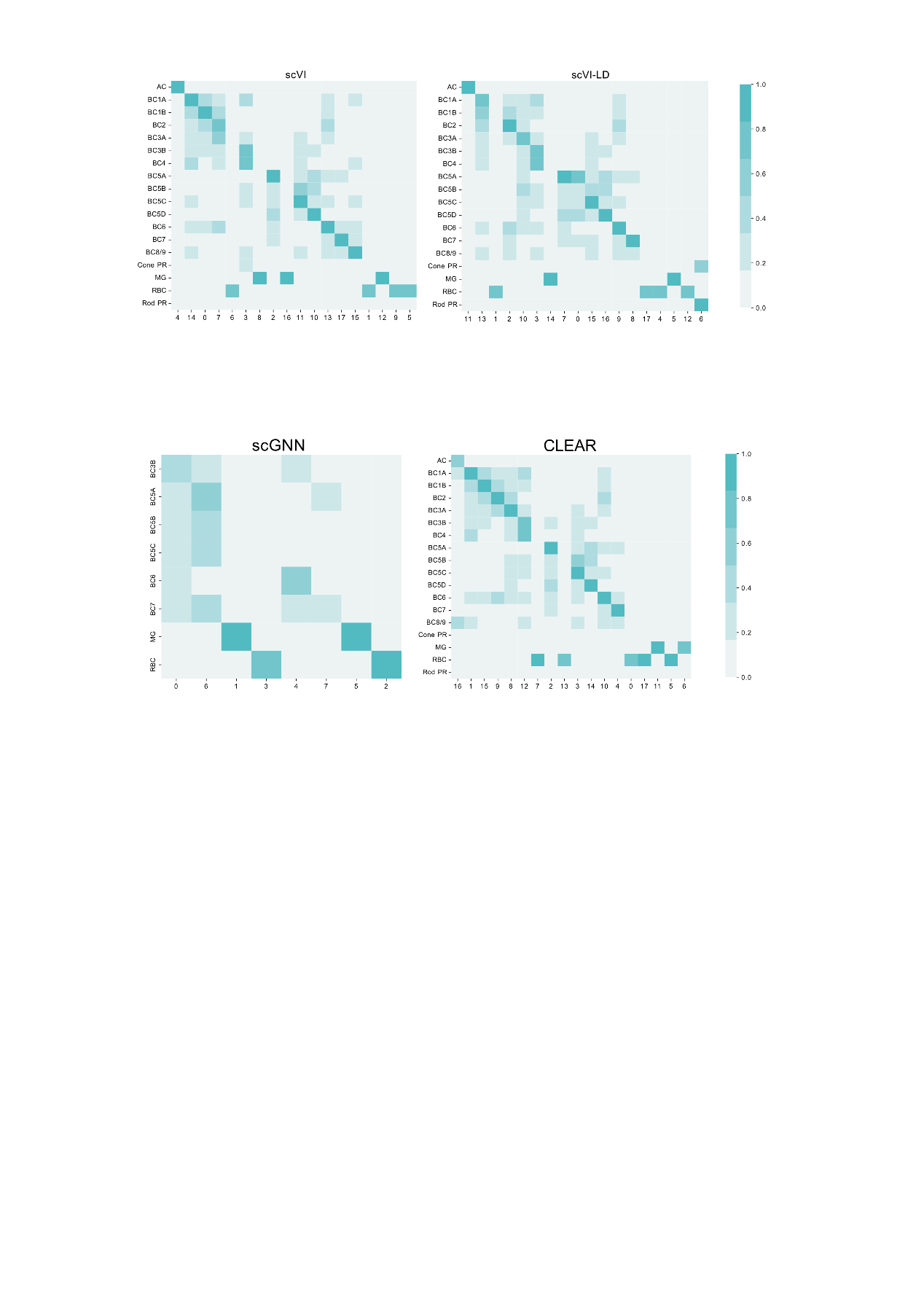


**Supplementary Figure 10.** Overlap of top 50 differentially expressed genes in clusters detected by scGNN and CLEAR with true cell types.


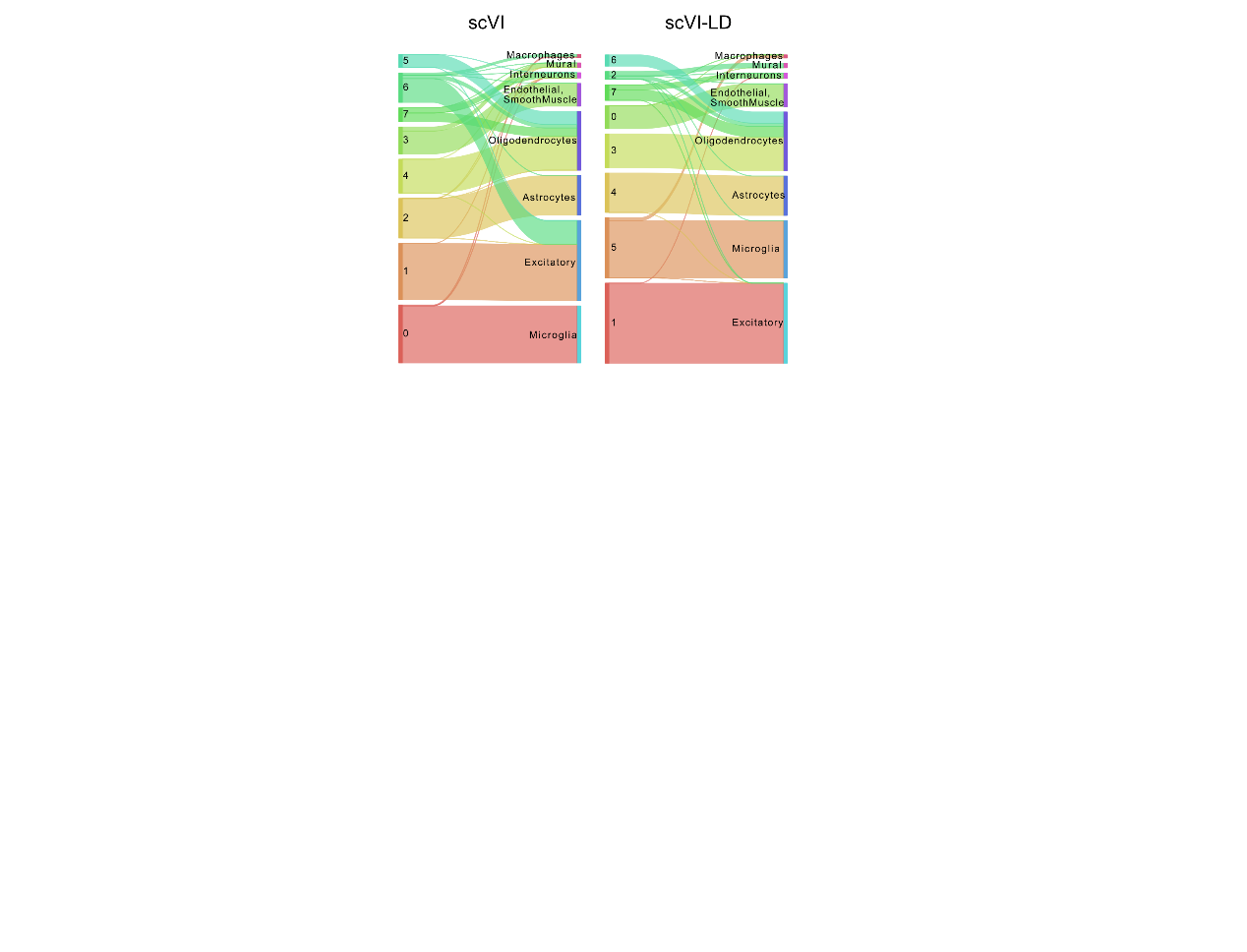


**Supplementary Figure 11**. Sankey plots of clustering results and true cell types for scVI and scVI-LD. For each subplot, the left side represents the clustering labels generated by each method, while the right side represents the true cell types.


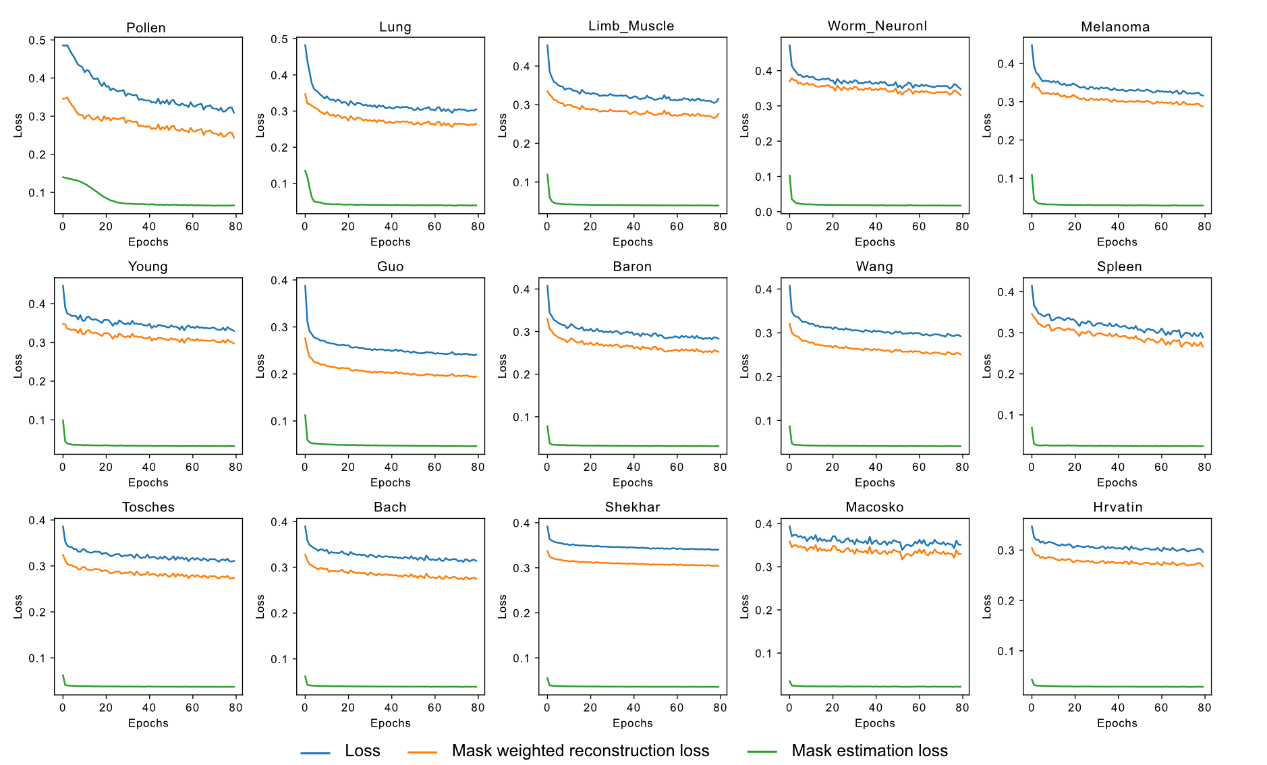


**Supplementary Figure 12.** The loss value over iteration on the 15 datasets.

*
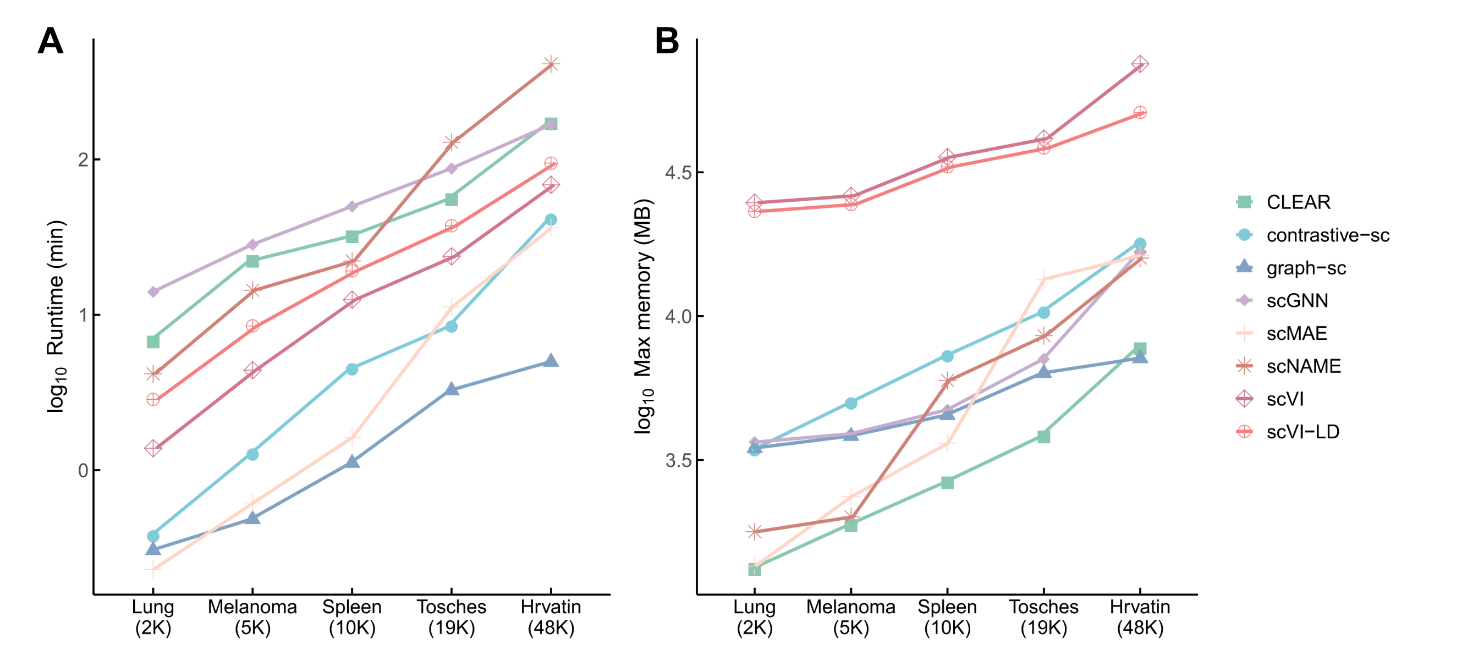
*

**Supplementary Figure 13. A.** Running times for scMAE and 7 comparsion methods. **B.** Memory consumption for scMAE and 7 comparsion methods.


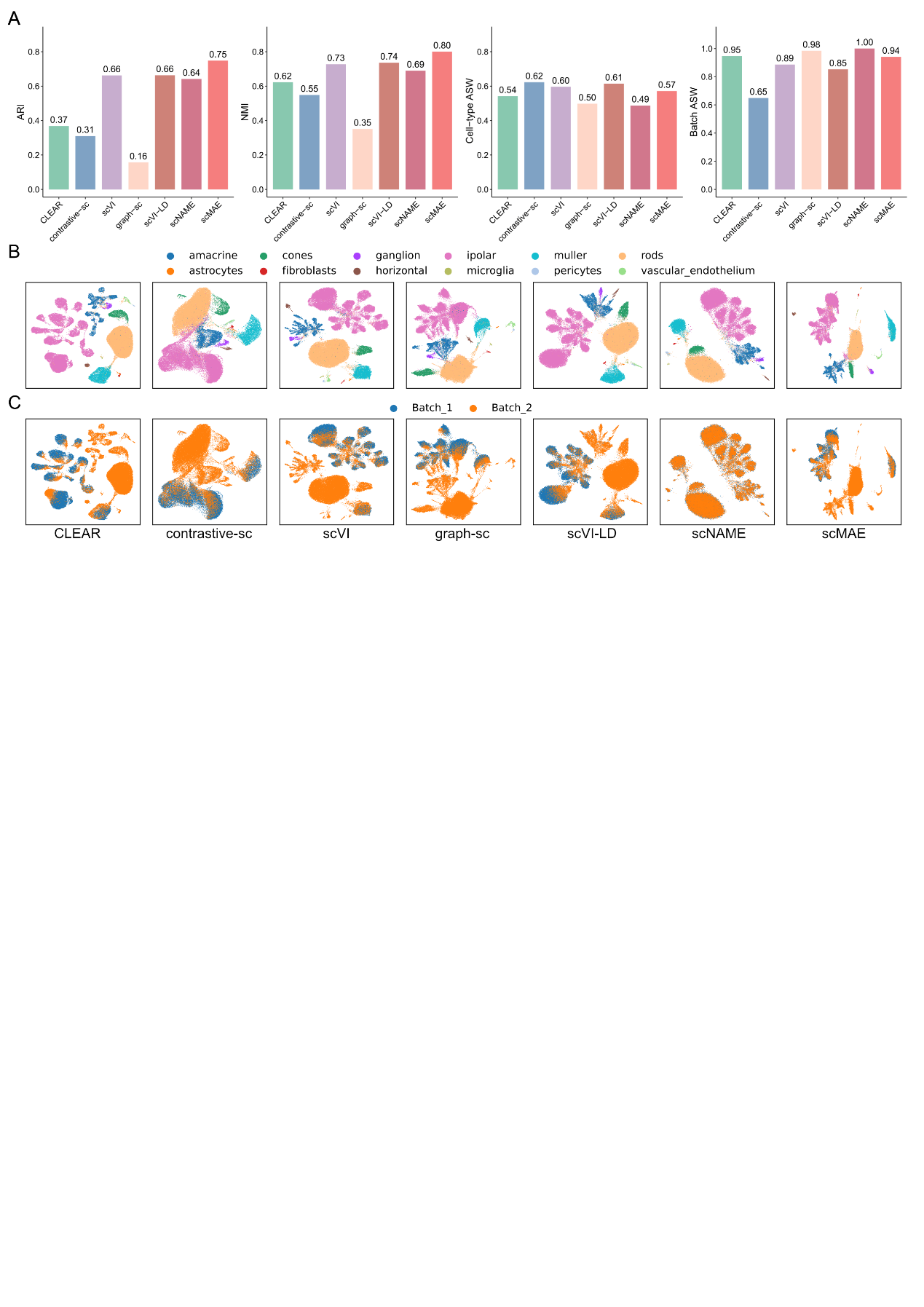


**Supplementary Figure 14.** **A**. Bar plots showing the ARI, NMI, Cell-type ASW and Batch ASW values on the MouseRetina dataset using scMAE and 6 comparsion methods. **B**. UMAP visualization of the cell embeddings learned by scMAE and 6 comparative methods. The colors represent the true cell types. **C**. The colors represent the batches.

**Supplementary Note 1. Details in data-preprocessing**

To improve the data quality, we filtered out low-quality cells with fewer than 1 genes and genes expressed in less than one cells. Then, we used scanpy to normalize each cell by total counts over all genes, and then log-transform the dataset. After that, 1000 highly variable genes were selected.

**Supplementary Note 2. Hyperparameter configuration of scMAE**

For each gene, we set a 0.4 probability of being masked.

The scMAE method is implemented using PyTorch in Python. The encoder module in scMAE has a hidden layer dimension of 256 and a latent embedding space dimension of 128. For the reconstruction loss, the weights assigned to masked data and unmasked data are 0.75 and 0.25, respectively. The weights of the reconstruction loss and mask estimation loss are set to 0.3 and 0.7, respectively. These loss functions are optimized using the Adam optimizer with an initial learning rate of 1e-3. The training process is conducted for a total of 80 epochs.

The experiments described in this paper were performed on a system running Ubuntu 18.04.6 LTS with an Intel (R) Core (TM) i9-10980XE CPU operating at 3.00 GHz and 64 GB of memory. To accelerate the training process, a GPU (GeForce RTX™ 3090Ti) was utilized.

**Supplementary Note 3. Clustering results of data with batch effects**

We used two datasets with batch effects, namely, MouseRetina and CellLine [14]. In addition to evaluating clustering results using metrics such as ARI, NMI, and Cell-type ASW [15], we calculate an ASW score on batches to assess the extent of batch effect removal. In this context, we scale and invert the ASW score for consistent metric comparison:

$$Batch\text{ }ASW=1-abs(ASW)$$

A higher final score indicates better mixing, reflecting improved batch removal effects.

The MouseRetina dataset, independently generated by two laboratories using Drop-seq technology, consists of two batches with varying cell counts and shared genes. The first batch comprises 26,830 cells, while the second batch includes 44,808 cells, both sharing 12,333 genes. However, these two batches only share half of the cell types. Unfortunately, during the execution of scGNN, an "out of memory" issue occurred, leading to the use of only scMAE and the other six comparative methods. In **Supplementary Figure 14A**, scMAE demonstrated optimal performance in clustering metrics ARI and NMI. Although the Batch ASW metric, indicating batch effect removal, may not be the highest, it still achieved a high score of 0.94. We further conducted UMAP visualization for the eight comparison methods, as shown in **Supplementary Figure 14B**, where different colors represent distinct cell types, and **Supplementary Figure 14C**, where different colors denote various batches. These results suggest that scMAE achieves satisfactory clustering results when handling data with batch effects.

**References**

[1] Pollen, A. A., Nowakowski, T. J., Shuga, J., Wang, X., Leyrat, A. A., Lui, J. H., Li, N., Szpankowski, L., Fowler, B., Chen, P., et al. (2014). Low-coverage single-cell mRNA sequencing reveals cellular heterogeneity and activated signaling pathways in developing cerebral cortex. Nature biotechnology, 32(10), 1053–1058.

[2] Tabula Muris Consortium, Overall Coordination, and Logistical Coordination (2018). Single-cell transcriptomics of 20 mouse organs creates a Tabula Muris. Nature, 562(7727), 367–372.

[3] Cao, J., Packer, J. S., Ramani, V., Cusanovich, D. A., Huynh, C., Daza, R., Qiu, X., Lee, C., Furlan, S. N., Steemers, F. J., et al. (2017). Comprehensive single-cell transcriptional profiling of a multicellular organism. Science, 357(6352), 661–667.

[4] Tirosh, I., Izar, B., Prakadan, S. M., Wadsworth, M. H., Treacy, D., Trombetta, J. J., Rotem, A., Rodman, C., Lian, C., Murphy, G., et al. (2016). Dissecting the multicellular ecosystem of metastatic melanoma by single-cell RNA-seq. Science, 352(6282), 189–196.

[5] Young, M. D., Mitchell, T. J., Vieira Braga, F. A., Tran, M. G., Stewart, B. J., Ferdinand, J. R., Collord, G., Botting, R. A., Popescu, D.-M., Loudon, K. W., et al. (2018). Single-cell transcriptomes from human kidneys reveal the cellular identity of renal tumors. science, 361(6402), 594–59.

[6] Guo, J., Grow, E. J., Mlcochova, H., Maher, G. J., Lindskog, C., Nie, X., Guo, Y., Takei, Y., Yun, J., Cai, L., et al. (2018). The adult human testis transcriptional cell atlas. Cell research, 28(12), 1141–1157.

[7] Baron, M., Veres, A., Wolock, S. L., Faust, A. L., Gaujoux, R., Vetere, A., Ryu, J. H., Wagner, B. K., Shen-Orr, S. S., Klein, A. M., et al. (2016). A single-cell transcriptomic map of the human and mouse pancreas reveals inter-and intra-cell population structure. Cell systems, 3(4), 346–360.

[8] Wang, Y., Tang, Z., Huang, H., Li, J., Wang, Z., Yu, Y., Zhang, C., Li, J., Dai, H., Wang, F., et al. (2018). Pulmonary alveolar type I cell population consists of two distinct subtypes that differ in cell fate. Proceedings of the National Academy of Sciences, 115(10), 2407–2412.

[9] Tosches, M. A., Yamawaki, T. M., Naumann, R. K., Jacobi, A. A., Tushev, G., and Laurent, G. (2018). Evolution of pallium, hippocampus, and cortical cell types revealed by single-cell transcriptomics in reptiles. Science, 360(6391), 881–888.

[10] Bach, K., Pensa, S., Grzelak, M., Hadfield, J., Adams, D. J., Marioni, J. C., and Khaled, W. T. (2017). Differentiation dynamics of mammary epithelial cells revealed by single-cell RNA sequencing. Nature communications, 8(1), 1–11.

[11] Shekhar, K., Lapan, S. W., Whitney, I. E., Tran, N. M., Macosko, E. Z., Kowalczyk, M., Adiconis, X., Levin, J. Z., Nemesh, J., Goldman, M., et al. (2016). Comprehensive classification of retinal bipolar neurons by single-cell transcriptomics. Cell, 166(5), 1308–1323.

[12] Macosko, E. Z., Basu, A., Satija, R., Nemesh, J., Shekhar, K., Goldman, M., Tirosh, I., Bialas, A. R., Kamitaki, N., Martersteck, E. M., et al. (2015). Highly parallel genome-wide expression profiling of individual cells using nanoliter droplets. Cell, 161(5), 1202–1214.

[13] Hrvatin, S., Hochbaum, D. R., Nagy, M. A., Cicconet, M., Robertson, K., Cheadle, L., Zilionis, R., Ratner, A., Borges-Monroy, R., Klein, A. M., et al. (2018). Single-cell analysis of experience-dependent transcriptomic states in the mouse visual cortex. Nature neuroscience, 21(1), 120–129.

[14] Tran H T N, Ang K S, Chevrier M, et al. A benchmark of batch-effect correction methods for single-cell RNA sequencing data[J]. Genome biology, 2020, 21: 1-32.

[15] Lotfollahi, M., Naghipourfar, M., Luecken, M.D. et al. Mapping single-cell data to reference atlases by transfer learning. Nat Biotechnol 40, 121–130 (2022).
